# Embodied Processing at Six Linguistic Granularity Levels: A Consensus Paper

**DOI:** 10.5334/joc.231

**Published:** 2023-10-10

**Authors:** Anita Körner, Mauricio Castillo, Linda Drijvers, Martin H. Fischer, Fritz Günther, Marco Marelli, Olesia Platonova, Luca Rinaldi, Samuel Shaki, James P. Trujillo, Oksana Tsaregorodtseva, Arthur M. Glenberg

**Affiliations:** 1 Department of Psychology, University of Kassel, DE; 2 Center for Basic Research in Psychology, University of the Republic of Uruguay, UY; 3 Max Planck Institute for Psycholinguistics, NL; 4 Department of Psychology, University of Potsdam, DE; 5 Department of Psychology, Humboldt-Universität zu Berlin, DE; 6 Department of Psychology, University of Milano-Bicocca, IT; 7 Department of Brain and Behavioral Sciences, University of Pavia, IT; 8 Department of Behavioral Sciences, Ariel University, IL; 9 Donders Institute for Brain, Cognition and Behaviour, Radboud University, NL; 10 Department of Psychology, University of Tübingen, DE; 11 Linguistic Anthropology Laboratory, Tomsk State University, RU; 12 Department of Psychology, Arizona State University, US; 13 Department of Psychology, University of Wisconsin-Madison, US; 14 INICO, Universidad de Salamanca, ES

**Keywords:** embodied cognition, grounded cognition, language, situated cognition

## Abstract

Language processing is influenced by sensorimotor experiences. Here, we review behavioral evidence for embodied and grounded influences in language processing across six linguistic levels of granularity. We examine (a) sub-word features, discussing grounded influences on iconicity (systematic associations between word form and meaning); (b) words, discussing boundary conditions and generalizations for the simulation of color, sensory modality, and spatial position; (c) sentences, discussing boundary conditions and applications of action direction simulation; (d) texts, discussing how the teaching of simulation can improve comprehension in beginning readers; (e) conversations, discussing how multi-modal cues improve turn taking and alignment; and (f) text corpora, discussing how distributional semantic models can reveal how grounded and embodied knowledge is encoded in texts. These approaches are converging on a convincing account of the psychology of language, but at the same time, there are important criticisms of the embodied approach and of specific experimental paradigms. The surest way forward requires the adoption of a wide array of scientific methods. By providing complimentary evidence, a combination of multiple methods on various levels of granularity can help us gain a more complete understanding of the role of embodiment and grounding in language processing.

The body plays a substantial role in language processing. When reading or hearing language, people tend to simulate sensations, movements, and emotions that are stated or implied by linguistic materials (for reviews, see Bergen, 2015; Fischer & Zwaan, 2008; Körner, Topolinski, & Strack, 2015; Meteyard et al., 2012; for theoretical explanations, see Barsalou, 1999, 2008; Glenberg & Gallese, 2012). These **simulations** (technical terms are defined in the glossary, see Table 1, and are bolded upon first mention) involve sensorimotor brain areas and body parts that are also active during the performance of the respective action or the sensory or emotional experience. For example, reading action words referring to hand, mouth, or foot action (e.g., *pick, lick*, or *kick*), has been found to increase brain activation in pre-motor and motor areas responsible for acting with the hand, mouth, and foot, respectively (Hauk, Johnsrude, & Pulvermüller, 2004). Reading emotional words has been found to involve facial muscle activity that accords with the implied emotion (e.g., zygomaticus activity for joy-related words and levator activity for disgust-related words; Niedenthal et al., 2009). Reading words referring to different sensory modalities, for example, visual, gustatory, or auditory words, has been found to increase activation in respective sensory brain areas (e.g., Goldberg, Perfetti, & Schneider, 2006). According to **embodied** cognition theories, these sensorimotor simulations are causally involved in conceptual processing.

**Table 1:** Explanations and Examples for the Employed Technical Terms.


TERM	EXPLANATION	EXAMPLE

**Amodal**	A representation (e.g., of meaning) that does not include activity in sensory, motor, or emotional cortices or bodily activity such as gestures.	Abstract concepts, such as *liberty*, are frequently conceptualized as representations devoid of sensorimotor aspects (however, see Borghi et al., 2019).

**Distributional semantic models**	A class of computational models based on the hypothesis that words with similar meanings have similar distributions over linguistic contexts. These distributions can be extracted from corpora of natural language.	The concepts *whale* and *dolphin* occur in highly similar linguistic contexts (with *sea, fish, ocean* as frequent words in close vicinity) and can therefore be assumed to have similar meanings.

**Embodied** (see also Grounded and Situated)	The second level in Fischer’s (2012) proposed hierarchy of knowledge representation. Sensorimotor associations resulting from previous experiences, such as actions and their outcomes	Right-handed people associate the right side in space with good and the left side in space with bad, whereas left-handed people show the opposite association (Casasanto, 2009).

**Grounded** (see also Embodied and Situated)	The first level in the hierarchy of Fischer (2012). Associations whose origins lie in the way the physical world is structured.	Accumulating objects cannot permeate one another, leading to associations of *more* with *up* (Lakoff & Johnson, 1980).

**Iconicity**	Non-arbitrary relations between sublexical elements of (spoken or signed) words and denoted concepts.	Association between high-frequency vowels (e.g., /i/ vs. /a/) and small (vs. large) size.

**Imagine manipulation** (see also Physical manipulation)	The second process taught to children (in Glenberg et al., 2004) to help them index words, phrases, and syntax to their meanings. When reading texts, children are asked to imagine moving images or toys into a configuration that accurately portrays the meaning of the text.	When reading that a farmer brings the cart to the barn, children would imagine the toy farmer moving to the toy cart and then both moving to the toy barn.

**Indexing**	The process of mapping a linguistic term onto its referent.	Mapping the word *horse* onto a toy horse.

**Physical manipulation** (see also Imagine manipulation)	The first process taught to children (in Glenberg et al., 2004) to help them index words, phrases, and syntax to their meanings. When reading texts, children are asked to physically move images or toys into a configuration that accurately portrays the meaning of the text.	When reading that a farmer brings the cart to the barn, children would move the toy farmer to the toy cart and then move both to the toy barn.

**Sensorimotor simulation**	In the context of language comprehension, sensorimotor simulation is activity in sensorimotor and emotional cortices in response to linguistic stimuli that is homologous to the activity engendered by the perception of the described objects, events, or activities.	When reading the word *salty*, gustatory brain regions would be active in a way similar to when tasting something salty.

**Situated** (see also Grounded and Embodied)	The third level in Fischer’s (2012) hierarchy. The human cognitive system is influenced by its current environment, adapting in a flexible manner to current goals and task constraints even when long-standing embodied or grounded knowledge conflicts.	One’s current state of high (vs. low) fatigue lead to hills being estimated as more (vs. less) steep (Bhalla & Proffitt, 1999). Depending on the number range (0–5 vs. 4–9), the numbers 4 and 5 are associated with either right or left space (Dehaene, Bossini, & Giraux, 1993).

**Symbol grounding-by-language**	Language can be conceptualized as experience-by-proxy (Johnson-Laird, 1983) or second-hand experience. Symbol grounding-by-language describes the idea that not only primary sensorimotor experience, but also this secondary experience can establish grounding for symbols.	For someone who has no direct sensorimotor experience with the concept *zebra*, reading that a zebra is a horse with black and white stripes will provide grounding for zebra. Thus, language alone will lead to a representation of the concept that incorporates sensorimotor information (Harnad, 1990).

**Symbol grounding problem**	The observation that language cannot be a self-contained system, in which each symbol is only defined by other symbols, as this would constitute an infinite regress of dependencies (Harnad, 1990).	Trying, as a thought experiment, to learn Chinese using nothing but a Chinese/Chinese dictionary would mean seeing unknown symbols when looking up an unknown symbol, leading to an infinite pass from symbol to symbol without gaining any understanding of Chinese (Harnad, 1990).


During sensorimotor simulations, body morphology and current sensorimotor states interact with the simulation, influencing concurrent processes. Thus, behaviorally, sensorimotor simulation is typically demonstrated by showing that manipulations of sensory or motor states influence linguistic tasks. For example, a concurrent task that alters facial expressions has been found to influence how words related to emotions are evaluated (Niedenthal et al., 2009) and to lead to faster responses to sentences implying similar (vs. different) emotions (Havas, Glenberg, & Rinck, 2007). Similarly, a concurrent manipulation of participants’ handshape has been found to facilitate (vs. impede) their understanding of sentences that imply congruent (vs. incongruent) hand actions (Klatzky et al., 1989).

From findings like these, embodied cognition theories conclude that the human mind cannot be understood as an **amodal** (see Table 1) information processing system (e.g., Glenberg, 2015). That is, modal information is not stripped from representations before central cognitive processing on this information takes place, and motor commands are not solely an output of cognitive processing. Some researchers have gone so far as to postulate that conceptual understanding requires simulation (Gallese & Lakoff, 2005), others hold the more moderate view that sensorimotor simulation aids or completes conceptual processing (Vigliocco et al., 2004), whereas still others hold that sensorimotor simulation is only an epiphenomenon, that is neither necessary nor helpful for conceptual processing (Mahon & Caramazza, 2008; for an overview, see Meteyard et al., 2012). The present work argues that sensorimotor simulation does play some functional (instead of epiphenomenal) role in conceptual processing. However, it does not subscribe to any specific embodied cognition theory. For a critical discussion of different theoretical positions, see Robinson and Thomas (2021); for an overview of predecessors and influences on embodied cognition theories, see Shapiro and Spaulding (2021).

According to theories of embodied and grounded cognition, much of cognitive processing is influenced by one’s current sensations (i.e., it is **situated**), by one’s body morphology and the resulting sensorimotor experiences (i.e., it is embodied), and by ecological properties of the world one lives in (i.e., it is **grounded**; Fischer, 2012; Fischer & Brugger, 2011; see also Fischer & Shaki, 2018; Myachykov et al., 2014; Pezzulo et al., 2013). It is worth noting that the proposed distinction between grounded, embodied, and situated knowledge is conceptual. In practice, behavior is typically jointly shaped by all three levels, as can be illustrated with the influence of finger counting on number processing: our evolutionary history determines the bimanual morphology with 5 fingers on each hand, which can explain the widespread usage of the decimal number system; while the acquisition of number knowledge begins with finger movements, which in turn depend on constraints such as the presence and location of interaction partners and objects and spatial associations of small (vs. large) numbers (e.g., Wasner et al., 2014).

In language processing, sensorimotor simulation can be examined at various levels of granularity, from phonemes as the smallest meaningful language level to corpora of texts as the largest granularity level. Thus, not only the processing of words but also the understanding of sentences and larger texts has been postulated to rely on the sensorimotor system. However, extrapolating findings across levels of granularity can be problematic (Zwaan, 2021), so that evidence for grounding in word processing cannot be seen as evidence for grounding in text processing. Therefore, we examine embodied and grounded cognition for each level separately before discussing the combined evidence.

The present consensus article is based on presentations at the 2021 Embodied and Situated Language Processing conference; specifically, these presentations focused on behavioral methods for the investigation of embodiment in language processing. This consensus article integrates these presentations as well as additional context information and expands the linguistic granularity levels. Thus, the article should be read as an assessment of the current state of the described phenomena and as an entry into the literature. It is not, however, a comprehensive review of the discussed granularity levels, nor does it discuss embodied and grounded cognition theories. Additionally, the present article passes over topics that are more fully covered in other contributions of this special issue, notably grounded, embodied, and situated cognition in language acquisition (see Reggin et al., 2022) and abstraction (Banks et al., 2022), as well as inter-individual differences (Ibáñez et al., 2022) and brain signatures in grounded, embodied, and situated cognition (Bechtold et al., 2022).

## Linguistic Granularity Levels

Early research on the influence of grounded, embodied, and situated processes on language processing examined mainly word level and sentence level processing. In more recent years, other granularity levels have also been examined. Here, we discuss research on six granularity levels; from a micro-level, examining how grounding influences word meaning; to the embodied and situated processing of words, sentences and texts; as well as the influence of situated processes in conversations; up to a macro-level, examining grounded, embodied and situated influences in text corpora.

### Sub-words

In linguistics, the mapping between word form and meaning has long been thought to be essentially arbitrary (e.g., Hockett, 1963). For example, using the term *language* to refer to a system of communication seems purely arbitrary—the same meaning might just as well be denoted by any other pronounceable combination of letters. However, various lines of research have demonstrated non-arbitrary associations between word form and meaning, called **iconicity** or sound symbolism (for reviews see, Dingemanse et al., 2015; Murgiano, Motamedi, & Vigliocco, 2021; Perniss, Thompson, & Vigliocco, 2010; Sidhu & Pexman, 2018). The most well-known iconicity phenomenon is the Bouba-Kiki effect—the phenomenon that, for most people, the pseudo-word *Bouba* fits as a name for rounded objects and the pseudo-word *Kiki* fits as a name for spiky objects (e.g., Ćwiek et al., 2021; Köhler, 1929). In general, language is more iconic than would be expected from random pairings of word form and meaning (Blasi et al., 2016; Monaghan et al., 2014; see also Winter et al., 2017). The origin of iconic associations is mostly unclear (Sidhu & Pexman, 2018). Some are thought to originate from statistical regularities that differ between languages (Bergen, 2004). However, for other iconicity phenomena in both spoken and signed languages, there is evidence of grounding or embodiment.

#### Iconicity in Sign Languages

Sign language phonology consists of three main components: handshape, movement, and location (Pfau, Steinbach, & Woll, 2012). Locations are arranged in front of the signer in a three-dimensional space, composed of three axes: horizontal, sagittal, and vertical (Emmorey, 2001). The location and movement in the visual three-dimensional space as well as the handshape provide a wide range of possibilities to express meaning through sign forms. As a consequence, sign languages have a large proportion of iconic signs; that is, signs and their sublexical components frequently depict some aspect of the concept they refer to (Baus, Carreiras, & Emmorey, 2013; Thompson, Vinson, & Vigliocco, 2009).

Many instances of imitative iconicity exist in sign languages. For example, in American sign language (Caselli et al., 2017) and Uruguayan sign language (Alisedo et al., 2007), for the sign *to drink*, the signer mimics drinking. However, some signs appear to be partially arbitrary. That is, one or two sublexical features represent the concept iconically while the remaining ones can be arbitrary. For example, in Uruguayan sign language and British sign language (Schembri et al., 2013), the sign *bike* is realized by handshape and movement mimicking the action of pedaling but the location is arbitrary. For still other signs—for example, *bad* or *nice* in British sign language—all sublexical features appear to be arbitrary.

An interesting group of concepts to examine for iconicity in sign languages are temporal concepts. As time is an abstract concept, imitative iconicity is impossible. However, time is metaphorically associated with space (e.g., Boroditsky, 2000; Lakoff & Johnson, 1980), in that time metaphorically moves on the sagittal axis with the past behind and the future in front (at least in Western cultures). This metaphoric association can stem from embodied experiences. When moving, humans typically move in the direction they are facing, so that objects behind are related to the past and objects in front are related to the future. Thus, in one’s ecological experiences, the sagittal spatial axis is related to deictic time.

A study on the Uruguayan Sign Language examined whether words representing temporal concepts use space in a way consistent with metaphoric associations between time and space (Castillo, Fojo, & Aguirre, 2021). The 97 temporal concepts registered in the Uruguayan sign language were classified into two categories. The first category was type of reference, consisting of, for example, concepts related to time tracking (e.g., *hour, minute*) and planetary events (e.g., *night, spring*). The second category was time construal, composed of deictic (e.g., *future, today*), sequential (e.g., *before, while*), and interval (e.g., *brief, period*) concepts (Núñez & Cooperrider, 2013). All signs for temporal concepts were then examined for movement direction. The results indicate that signs describing deictic time concepts more frequently use the sagittal axis compared to the other two axes, consistent with the *past is back and future is forward* metaphor (Castillo, Fojo, & Aguirre, 2021). Similar associations have been observed in other sign languages. In many Western sign languages, for example, signs like *yesterday* or *past* use a backward movement and *future* or *tomorrow* use a forward movement (Sinte, 2013).

In sum, in the time lexicon of the Uruguayan and other sign languages, spatial patterns in the sagittal axis systematically represent temporal semantic constructs, consistent with the metaphoric association between time and the sagittal axis in space. Thus, although imitative iconicity is very salient in sign languages, subtler forms of iconicity also exist. These associations between word meaning and sublexical sign features can be explained as embodied, originating in ecological experiences of moving through time and space.

#### Iconicity in Spoken Languages

In spoken languages, several form–meaning associations have been argued to be grounded or embodied (e.g., Vainio & Vainio, 2021). Grounding is especially salient in ideophones, a class of iconic words that are characterized by an overlap of sensory features between word form and meaning (for a review, see Dingemanse, 2012). For example, reduplication, that is, the partial or complete repetition of word parts, signals meaning, often a high intensity or a long duration. In Japanese, for example, the term *goro* denotes a heavy object rolling once, and the term *gorogoro* denotes a heavy object rolling continuously or repeatedly (Kita, 1997). Thus, the duration when hearing or articulating a word correlates with the event duration, suggesting the length of the word form is grounded in the ecological event duration.

Compared with ideophones, the grounding of other iconicity phenomena is more subtle. For instance, size sound symbolism, that is, the observation that words containing /i/ fit small objects and words containing /a/ fit large objects (Sapir, 1929), can be explained by auditory frequencies (Ohala, 1994). Specifically, small compared to large objects (e.g., small vs. large pipes) typically elicit higher pitched sounds. Additionally, vowels differ in frequencies (fundamental and formant frequencies; e.g., Whalen & Levitt, 1995). The associations between size and vowels conforms with their relative frequencies, so that vowels with high formant frequencies (e.g., /i/ and /e/) are associated with small size while vowels with low formant frequencies (e.g., /o/ and /a/) are associated with large size (Thompson & Estes, 2011; see also Blasi et al., 2016). Thus, vowel frequency, an auditory word feature, is associated with an auditory object feature encountered in the ecological environment (Parise & Spence, 2012). Accordingly, size sound symbolism can be explained by grounding.

Another iconicity phenomenon that rests on vowels is valence sound symbolism. Specifically, the vowel /i/ has been found to be associated with positive valence, while /o/, /u/ and /˄/ have been found to be associated with negative valence (Garrido & Godinho, 2021; Rummer et al., 2014; Rummer & Schweppe, 2019; Yu, McBeath, & Glenberg, 2021). Whereas size sound symbolism seems driven by grounding (ecological associations between auditory and vowel frequencies), valence sound symbolism seems rather driven by embodiment. Specifically, the association between vowels and valence has been found to result from overlapping muscle activity for emotional facial expressions and motor activity during articulation (Körner & Rummer, 2022). Both smiling and the articulation of /i/ involve a retraction of the lip corners and this muscle overlap explains the association between positive valence and the vowel /i/. In contrast, the articulation of vowels associated with negative affect frequently involves lip rounding, and this antagonistic muscles to the ones responsible for lip corner retraction (Körner & Rummer, 2022). Thus, valence sound symbolism can be explained as an embodied association, driven by the affective meaning of articulatory movements.

In sum, although iconicity is typically not examined from an embodied perspective (cf. Vainio & Vainio, 2021), several phenomena can be explained in terms of grounded or embodied experiences. Word form features can overlap with ecological features (e.g., object sound frequencies and speech sound frequencies) or with embodied features (e.g., articulation muscle tension and facial expression muscle tension); moreover, grounded experiences can lead to the usage of sublexical features that are associated with conceptual meaning (e.g., sagittal axis for deictic time). These phenomena demonstrate how, over the course of language development, human experiences influenced language. Words where linguistic features are congruent with grounded or embodied experiences emerged and persisted (perhaps because systematic symbol–meaning associations are easy to learn; see e.g., Imai & Kita, 2014), leading to an association between word elements and grounded or embodied experiences. As a result, the influence of embodied or grounded processes can be detected in the spoken and signed words we use.

### Words

From the embodied cognition perspective, word understanding partially relies on modality-specific simulations, involving sensorimotor brain areas that are active when perceiving real objects or performing real actions (e.g., Hauk, Johnsrude, & Pulvermüller, 2004; Pecher, Zeelenberg, & Barsalou, 2003). For example, the word *red* denotes a quality perceived through sight, and hence its understanding might involve visual brain areas. Many behavioral studies, using a wealth of different paradigms, support simulations playing a part in conceptual processing. However, some studies in embodied cognition find no effect (e.g., Petrova et al., 2018) or inconsistent effects (see Shebani & Pulvermüller, 2018, for a discussion). By now, several boundary conditions have been observed for various phenomena. The most prominent boundary conditions seem to be related to (a) timing, such as the time lag between different stimuli (e.g., Boulenger et al., 2006; Estes & Barsalou, 2018; García & Ibáñez, 2016); and (b) type of task, with tasks involving semantic compared to lexical processing facilitating embodied simulation effects (Günther, Nguyen et al., 2020, Scerrati et al., 2017). We discuss this evidence here in more detail.

#### The Modality Switch Effect

Simulation during word reading is hypothesized to be related to the implied sensory or motor modality. This hypothesis has been tested by comparing modality repetitions with modality switches. During reading, when the perceptual modality changes (e.g., the word *red*, implying the visual modality, follows the word *loud*, implying the auditory modality), cognitive resources are needed to switch attention from one modality to another, leading to longer processing latencies—a phenomenon known as the modality switch effect (Pecher, Zeelenberg, & Barsalou, 2003). Just like perception (see Lukas et al., 2010, for a review), language comprehension requires cognitive resources when redirecting attention from one modality to another. To examine the linguistic modality switch effect, typically the property verification task is used. In this task, participants read short sentences (e.g., BLENDER can be LOUD) and are asked to verify whether the property (here: LOUD) is typical for the concept. The modality switch effect consists in longer verification times when the evaluated property in the preceding trial was from a different modality (e.g., gustatory; e.g., CRANBERRIES can be TART) compared to the same modality (here, also auditory; e.g., LEAVES can be RUSTLING; Pecher, Zeelenberg, & Barsalou, 2003; see also Hald et al., 2011; Lynott & Connell, 2009; Pecher, Zeelenberg, & Barsalou, 2004; Scerrati et al., 2015; for the influence of both, language statistics and simulation, see Louwerse & Connell, 2011). This result suggests that modality information is activated during semantic word processing.

To examine whether the modality switch effect depends on semantic processing, some studies have used a lexical decision task that does not require semantic processing. Typically, the modality switch effect was reduced with the lexical decision task (e.g., Kuhnke, Kiefer, & Hartwigsen, 2020; Scerrati et al., 2017). However, if modality-specific processing starts early and is only brief (see Pulvermüller, Shtyrov, & Hauk, 2009), then the time gap between the two words needs to be as small as possible to enable detecting modality switch costs. A recent study presented stimuli simultaneously instead of sequentially, so that participants had to process two words at once (Platonova & Miklashevsky, 2022). Specifically, participants performed a lexical decision task on pairs of Russian adjectives (here, translated into English), one adjective above and the other below a central fixation cross (word + word, e.g., *warm* + *fuzzy*; or word + pseudo-word, e.g., *yellow* + *kemily*). The task was to distinguish cases when both stimuli were words from cases when at least one stimulus was a pseudo-word. The adjectives were visual (e.g., *white*), auditory (e.g., *quiet*), or haptic (e.g., *warm*).

**Figure 1:**
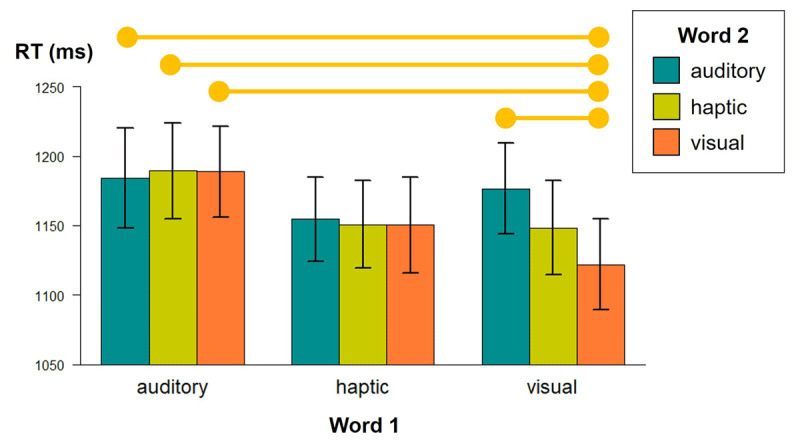
Mean reaction times for 9 conditions (combinations of semantic modalities: Word 1 × Word 2; see main text for details). Whiskers represent standard errors. Orange horizontal lines represent significant differences between conditions (*p* = .02 or lower).

This study observed that when the first (top) word implied the visual modality, reaction times differed depending on the implied modality of the second (bottom) word. The combination ‘visual + visual’ yielded faster reaction times than ‘visual + auditory’ or the combination of auditory with any other modality. No significant differences were found between combinations of visual and haptic modalities, see Figure 1 (Platonova & Miklashevsky, 2022). This study demonstrates that even surface lexical processing can lead to perceptual simulation when the two words have to be evaluated together and the time between processing different stimuli is minimal.

#### Word–Color Interferences

The simulation view suggests that characteristic object colors are also simulated when reading. For example, reading about a stop sign re-activates the perception of the stop sign and hence experiences involving the color red. This should hold even when the linguistic stimulus does not explicitly refer to the color. Experimentally, typical object color (e.g., red for the word *raspberry*) and response button color have been found to lead to congruency effects (Tsaregorodtseva et al., 2022). That is, responses were faster when the two colors matched compared to mismatched. This was observed both for individual words and for sentences, using lexical decision tasks and sentence sensibility judgments. However, when adding filler objects without any particular color (e.g., *car*) or with unrelated colors (e.g., *honey*), thereby decreasing the proportion of relevant items, congruency effects only occurred when isolated words were presented but not when sentences were presented (Tsaregorodtseva et al., 2022). This suggests that comprehenders indeed simulate color experiences when processing linguistic stimuli that refer to objects with a typical color (hereafter *color effect*). However, the study results showed that the effect was context sensitive and depended on the proportion of items related to visually presented colors.

This dependence on task aspects and context factors accords with previous findings from other experimental paradigms. Words associated with a specific color have been found to direct attention towards other objects depicted in the same color but not if the pictures were gray-scaled or when only words were presented (Huettig, Guerra, & Hero, 2020). Similarly, Yee, Ahmed, and Thompson-Schill (2012) observed the color effect only when participants had previously performed a Stroop-task. Thus, the color effect seems to require a task context in which visual colors are important (see also Huettig & Altmann, 2011; Mannaert, Dijkstra, & Zwaan, 2017).

A question that has not received much attention concerns the role of the linguistic context for the color effect. Connell and Lynott (2009) found that referring to objects with an atypical but possible color (e.g., bears at the North Pole) activated both the typical and the atypical color (brown and white).^1^ However, it is unclear to which extent such situated properties established by the linguistic context can influence the color effect. Moreover, it is not perfectly clear yet why some studies on the color effect show facilitation effects, whereas others find interference effects (Connell, 2007; Zwaan & Pecher, 2012; Naor-Raz, Tarr, & Kersten, 2003; for a more general discussion of facilitation vs. interference in sensorimotor simulation studies, see Connell & Lynott, 2012). Thus, the question about moderators and boundary conditions for the simulation of color experiences is far from resolved, although it seems clear that color simulation is context-dependent and flexible rather than stable.

#### Word–Space Associations

Sensorimotor simulations are generally thought to be rich, encompassing not only essential object properties, such as shape, but also object properties related to typical experiences with these objects, such as spatial location. Whether reading directs attention to typical object locations can be studied with a spatial probe task imported from attention research. In this task, first a lexical concept is presented at central fixation as an attention cue. Then, participants are asked to respond to a probe which can occur at many different spatial locations. Probe detection speed indicates whether attention was at the probed location (valid cue, fast responses) or not (invalid cue, slow responses; Posner, 1980).

The Posner paradigm has been extended to semantic cueing. For example, reading the word *left* (vs. *right*) has been found to lead to faster detection of probes on the left (vs. right) side of the screen (Hommel et al., 2001). This task combines good experimental control over relevant processes (such as time course) with high internal validity (i.e., measuring the intended construct). Interestingly, this semantic cueing method works not only with concrete concepts (e.g., *cloud* vs. *foot* for vertical locations; Gozli, Chasteen, & Pratt, 2013) but also with abstract concepts without concrete spatial experiences. For example, words like *god* (vs. *devil*) have been found to cue vertical attention, leading to faster probe detection in the upper (vs. lower) visual field (Chasteen, Burdzy, & Pratt, 2010). However, in some experimental settings, interference (instead of facilitation) effects have been observed, such that *head* (vs. *foot*) led to slower discrimination of upper (vs. lower) visual field probes (Estes, Verges, & Barsalou, 2008). Moreover, just like the previously reviewed effects, these attentional probe phenomena depend on a tight timing between prime and probe (Estes & Barsalou, 2018), and timing can also moderate whether facilitation or interference occurs (Gozli, Chasteen & Pratt, 2013).

Another class of abstract concepts known to have associations with space is numbers. Smaller (vs. larger) numbers from a given number range are associated with left (vs. right) space (the SNARC effect: spatial-numerical association of response codes; Dehaene, Bossini, & Giraux, 1993; McCrink, Dehaene, & Dehaene-Lambertz, 2007; Mioni, Fischer, & Shaki, 2021; Shaki, Pinhas, & Fischer, 2018). A word class that is related to numbers is quantifiers such as *many, few, usually* or *seldom*. In two recent studies with quantifiers, the SNARC effect was extended to SLARC (spatial-linguistic association of response codes; Dooley, 2021; Abbondanza et al., 2021). Dooley (2021) contextualized single quantifier words in winning or losing contexts and found the typical response side association for quantifiers representing smaller/larger values. For example, words related to large (vs. small) numbers, such as *many* (vs. *few*), led to faster responses with the right (vs. left) response keys, and this can be interpreted as reflecting corresponding attentional shifts (Dooley, 2021; for support from a different experimental paradigm, see Abbondanza et al., 2021).

Among the linguistic granularity levels, the word-level is probably the most well-examined level. In addition to sensory properties, such as color and space, also emotional (e.g., Havas et al., 2007) and motor properties (e.g., Frak et al., 2010) have been examined. Accordingly, the current state of knowledge concerning replicability and boundary conditions is more advanced for word-level phenomena than most other levels (an exception is the Action Sentence Compatibility effect, see next section). While some boundary conditions, for example large (vs. small) time lags between stimuli, have been observed for many word-level phenomena, others might be specific to some experimental paradigms or the simulated sensorimotor property. For example, results concerning color simulation seem to suggest that the sensory simulation of typical object color during conceptual processing occurs only under specific conditions, such as attention to color (see Yee, Ahmed, & Thompson-Schill, 2012) and high proportion of task-relevant colors (Tsaregorodtseva et al., 2022). As far as we know, there is almost no research that systematically compares the simulation of different sensorimotor properties (however, see de Koning et al., 2017). Accordingly, our observation that, compared to the simulation of other sensorimotor properties, color simulation might have narrower boundary-conditions and therefore might be a less automatic feature of sensorimotor simulations, is highly speculative and needs to be empirically tested.

### Sentences

The embodied simulation approach to language postulates sensorimotor simulation not only for isolated words but also for phrases and sentences. For example, when reading a sentence such as *You and your lover walked hand in hand on the moonlit tropical beach*, reading the phrase *moonlit tropical beach* activates a state in the visual system that is similar to actually seeing a moonlit tropical beach (Rueschemeyer et al., 2010); reading *walk* activates a state in the motor system that resembles actual walking (Hauk, Johnsrude, & Pulvermüller, 2004), and simulation in the emotional system helps understanding what it means to be holding hands with your lover (Havas et al., 2010). As these examples already show, simulation in sentence processing has been demonstrated for several sensorimotor properties, for example, for visual properties (Rueschemeyer et al., 2010; Horchak & Garrido, 2022; however, see also Ostarek et al., 2019; for differing evidence for visual simulation, depending on visual property, see de Koning et al., 2017) and emotional properties (Havas et al., 2007, 2010; for an overview of evidence for simulation on the sentence-level, see Horchak et al., 2014).

Here we focus on the action-sentence compatibility effect (or ACE; Glenberg & Kaschak, 2002) for two reasons. First, it was one of the first behavioral demonstrations of the role of action systems in language comprehension. Second, the effect is widely cited (Google Scholar lists over 2700 citations as of April, 2022) in both positive and negative contexts. To produce an action-sentence compatibility effect, participants read a sentence that implies action in one direction (e.g., toward the body as in *Courtney handed you the notebook*) or another (e.g., away from the body, as in *You handed Courtney the notebook*) and indicate whether the sentence is sensible by literally moving their hand toward or away from the body. The action-sentence compatibility effect is a congruency effect between implied action direction and literal response direction; congruent compared with incongruent directions result in faster responses. However, several failures to replicate the effect (e.g., Papesh, 2015; Morey et al., 2022) raise the question of whether this procedure is useful for investigating sentence simulation.

**Figure 2:**
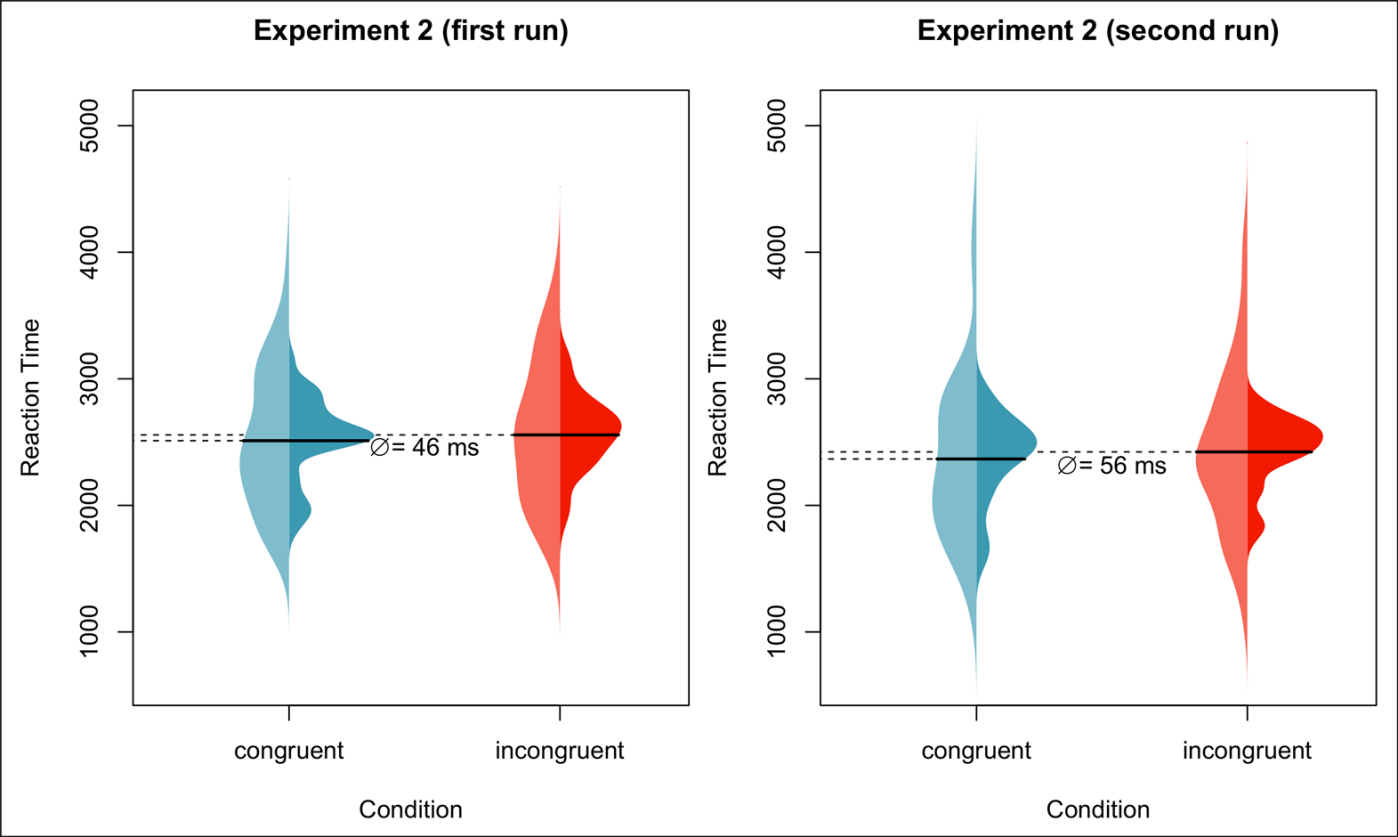
Data from Günther, Nguyen et al. (2020). Distribution over participants (lighter left part) and items (darker right part). The solid lines indicate the mean values. Reprinted from the *Journal of Memory and Language*, Volume 115, Günther, Nguyen, Chen, Dudschig, Kaup, & Glenberg, Immediate sensorimotor grounding of novel concepts learned from language alone, 2020, with permission from Elsevier.

Günther, Nguyen et al. (2020) demonstrated the usefulness of the action-sentence compatibility effect provided that certain constraints are met. Günther, Nguyen et al. (2020) used the effect to investigate whether concepts newly learned through language alone are immediately embodied. Participants learned new words and definitions (e.g., in one condition, *A mende is a head in which a very thin net of electrodes is implanted*, or in another condition, *A mende is a biomechanical foot that was initially developed to replace amputated feet*.) After learning the words, participants judged the sensibility of sentences such as, *You scratch your mende*. Note that for one learning condition, this sentence implies an upward motion, and for the other learning condition, the sentence implies a downward motion. To respond, participants literally moved their hands upward or downward. Congruency (vs. incongruency) of literal hand movement direction and implied sentence direction led to faster reaction times, replicating the action-sentence compatibility effect (Günther, Nguyen et al., 2020). Data from two independent, high-powered runs of the experiment are in Figure 2. These results demonstrate that the action-sentence compatibility effect can be useful for investigating new questions regarding the necessary and sufficient conditions for embodied cognition signatures to occur.

Over the course of several decades, three important constraints on the action-sentence compatibility effect have been identified, see Table 2. First, the effect size is small, for example, *d* = 0.14 and *d* = 0.15 for the two replications in Günther, Nguyen et al. (2020). Second, sentence perspective is important. For sentences in the third person, no action-sentence compatibility effect is observed unless the comprehender’s location within the event is available (Gianelli et al., 2011). Third, similar to word-level processing, the relative timing between sentence processing and responding is critical (Borreggine & Kaschak, 2006; Kaschak & Borreggine, 2008; de Vega, Moreno, & Castillo, 2013). In fact, in a meta-analysis, Winter, Dudschig, and Kaup (2021) report a significant positive average action-sentence compatibility effect (*d* = 0.21) with brief delays; however, with longer delays, the effect was significantly reversed (*d* = –0.14).

**Table 2:** Boundary Conditions for the Action-Sentence Compatibility Effect.


NUMBER	CONSTRAINT	SAMPLE PUBLICATION

1	Sufficient sensitivity to detect small effects	Günther, Nguyen et al. (2020)

2	Sentence perspective: 1^st^ person or comprehender’s location available	Gianelli et al. (2011)

3	Delay between reading and responding: Short	Borreggine & Kaschak (2006)


With these constraints in mind, let us examine some of the failures to replicate the action-sentence compatibility effect. Papesh (2015) was among the first to publish a non-replication. One may question the usefulness of this report, however, because the perspective (constraint 2) is not clear in many of those experiments, and because large effect sizes were assumed both in the power analyses and in the Bayesian meta-analysis, contrary to constraint 1. In contrast to what Papesh reports, if one performs the meta-analysis using a small effect size as a Bayesian prior, then the data are more consistent with a small action-sentence compatibility effect than with the null hypothesis.

**Figure 3:**
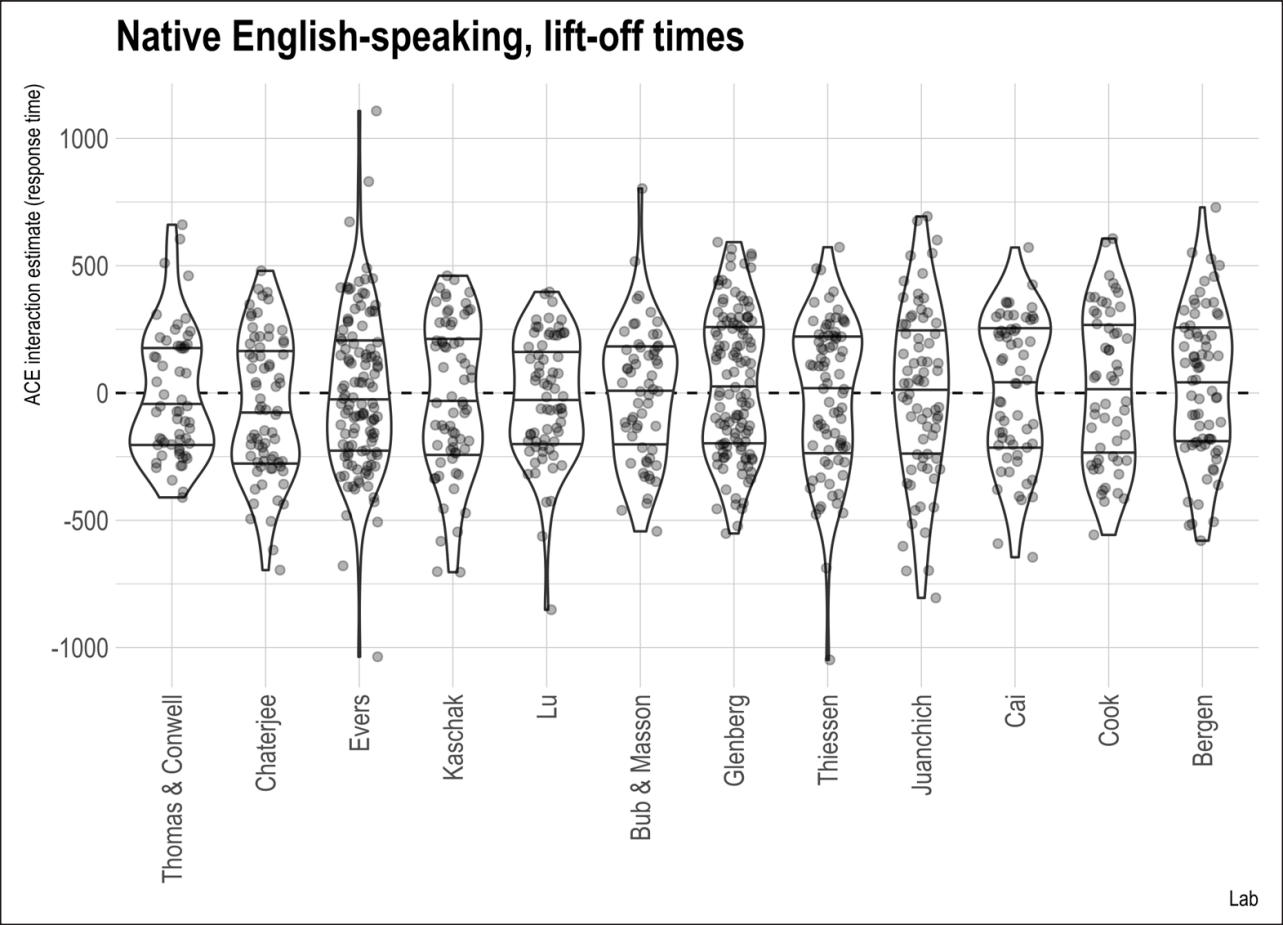
Data from Morey et al. (2021). Within each lab, the middle horizontal line indicates the median effect, and the two other lines indicate the inter-quartile range. Points are translucent, meaning that darker areas indicate overlapping points and thus higher density. Reprinted under the Creative Commons license (CC BY 4.0).

Another failure to replicate the action-sentence compatibility effect was reported by Morey et al. (2021). This registered replication attempt involved 18 labs and over 1000 participants, so there was sufficient statistical power to detect a small effect. Nonetheless, no action-sentence compatibility effect was found. Data from the predominantly English-speaking labs are shown in Figure 3. Although on average, the effect was close to zero, note that almost every lab shows a bi-modal distribution, that is, there is evidence for a positive action-sentence compatibility effect (a bulge in the upper part of the violin plot) and a negative action-sentence compatibility effect (a bulge in the lower part). The bi-modality was statistically significant, but why are there bi-modal effects?

The procedures used in Morey et al. were complex. First, the procedure was a go/no-go task so that the participant had to decide whether to respond on every trial. Second, the direction of response (toward versus away) was signaled for each trial and could change from trial to trial. Third, the sentences were presented aurally. Thus, there were many opportunities for participants to adjust how they responded: Should I be biased to go or to no-go; should I listen and comprehend the sentence before figuring out the response direction for a “sensible” response, or should I prepare the response first and then comprehend the sentence; how much of the sentence do I need to listen to before deciding? The speculation, then, is that some participants adjusted their performance so that relative timing of comprehension and responding resulted in a positive action-sentence compatibility effect, and some adjusted their performance so that the relative timing resulted in a negative action-sentence compatibility effect (constraint 3).

For the future, it appears that the action-sentence compatibility effect may be useful, but not as useful as originally thought. That is, when designing experiments, researchers need to keep in mind the three constraints (and others may yet be found) to ensure that this tool leads to reproducible findings.

### Understanding Discourse and Texts

If embodied simulation underlies language processing in general, then it should also underlie the understanding of units of language larger than words or sentences, such as stories. Of course, an important component of understanding discourse and texts is understanding the meaning of sentences. But texts are more than a collection of sentences because they are organized: Sentences repeat words to show related concepts, successive sentences expand on ideas, and they set up problems and later provide solutions. These organizations have variously been modeled as scripts (e.g., Schank & Abelson, 1977), story grammars (Thorndyke, 1977), and mental models (Johnson-Laird, 1983; Zwaan & Radvansky, 1998). Several research programs have addressed this organization from an embodied perspective including Berenhaus, Oakhill, and Rusted (2015), and Horchak, Giger, and Pochwatko (2014).

To the extent that embodied processes such as simulation are important for understanding texts, interventions that teach children simulation might improve children’s reading comprehension of stories. Toward that end, Glenberg and his colleagues have developed and tested several versions of a simulation intervention to improve the reading comprehension of young children. The basic idea is that comprehension results from simulation: reading event descriptions induces states in sensorimotor and emotional systems that are homologous to the states that result from experiencing the event. For example, reading a sentence such as *The farmer pulled the cart to the barn* can generate activity in the visual system that is similar to activity when actually seeing a farmer and a cart (Rueschemeyer et al., 2010) or actually pulling a cart (Hauk, Johnsrude, & Pulvermüller, 2004). Successful readers create these simulations easily, and perhaps they do not even consciously experience any visual or motor imagery. But young children, particularly those who can decode the words but do not comprehend well (e.g., Oakhill, Cain, & Bryant, 2003), might have deficits in simulations and would profit from being taught how to simulate while reading.

In the earliest research that attempted to use principles of embodied cognition as an intervention (e.g., Glenberg et al., 2004), first- and second-grade children were taught various types of imagery by using toys. For example, one set of toys included a toy barn, tractor, cart, farmer, etc. While reading a text about activities on a farm, when the child came across the sentence about the cart, she would move the toy farmer to the cart, and then move the farmer and the cart to the barn. Thus, the children practiced **indexing** (Glenberg & Robertson, 1999) nouns such as *farmer* to the toy farmer using the visual system. They also practiced indexing syntax (i.e., who does what to whom) to their own movements. Glenberg et al. called this activity **physical manipulation**. Compared to reading without manipulation (although with the toys visible), physical manipulation improved reading comprehension, measured by verbatim and inference questions, often with large effect sizes. Unfortunately, there was no transfer effect. That is, when using new texts without toys, previous use of physical manipulation did not improve reading comprehension.

The lack of transfer is understandable given that the children were not given any training in how to simulate on their own. To understand that training, consider that simulation can be an automatic process, particularly for skilled readers, whereas imagery is a deliberate process. Nonetheless, because deliberate imagery is very similar to simulation, it is likely to facilitate simulation (e.g., Jeannerod, 2001). In fact, imagery instructions had previously been found to aid text comprehension and memory (e.g., Gambrell & Jawitz, 1993). Thus, children in Glenberg’s research were given a deliberate imagery instruction called **imagine manipulation**. That is, after teaching children to use physical manipulation, they were then asked to imagine moving the toys, without actually touching them. Consistent with previous data (e.g., Gambrell & Jawitz, 1993), when children were taught to use imagine manipulation, the children, particularly those who were good decoders but needed help with comprehension, showed remarkably good transfer (Glenberg et al., 2004).

A clear demonstration of the effectiveness of training physical manipulation and then imagine manipulation comes from Adams, Glenberg, and Restrepo (2019). In this research, the children were native Spanish speakers living in the Canary Islands. Spanish has a transparent orthography, in which each letter is pronounced the same way in virtually all contexts. Thus, most typically developing Spanish children are good decoders, although not necessarily good comprehenders. For the research in the Canary Islands, children first read one set of stories using physical manipulation by manipulating images on a computer screen (rather than real toys); in the control condition, children read without manipulating the images (although the images were visible). The children who used (vs. did not use) the physical manipulation strategy showed much better comprehension with a large effect size \[(\eta _p^2 = 0.28)\]. The children then practiced imagine manipulation on different texts, but from the same scenario (e.g., other farm stories). Reading with (vs. without) imagine manipulation again resulted in a large reading comprehension benefit. Finally, all children read a transfer story from a new scenario (e.g., a story about a family living in a house). Again, children who had (vs. had not) practiced imagine manipulation in previous stories were found to have much better reading comprehension. Thus, teaching beginning readers to practice imagine manipulation led to their transferring their skills to new texts, resulting in markedly improved reading comprehension.

The future of this application of embodiment and situatedness looks promising. First, physical manipulation can be effective even without toys or technology: It is getting children to simulate that is important. Gómez and Glenberg (2022) report that when children used pantomime, in lieu of moving images on an iPad screen, results were almost identical to when children manipulated the images. Second, effects of physical manipulation and imagine manipulation are being adapted for languages other than English and Spanish. One project is being conducted in Shanghai to determine if physical manipulation and imagine manipulation are effective in teaching Mandarin-speaking children how to read for comprehension in English. Third, and perhaps most importantly, the training is being adapted for a web-based system that will make it easier and less expensive to both conduct research around the world as well as to make the benefits of embodiment-based training widely available to children, parents, and schools. In short, teaching children to use simulation to improve reading comprehension demonstrates that applying principles of embodied cognition can have enormous benefits in real-world tasks.

### Conversations

Beyond single words, sentences, and texts, grounding and embodiment also influence natural conversations; specifically multimodal cues influence face-to-face conversations and turn-taking. Studies from conversation analysis have long shown that the body plays an important role in social interaction (e.g., Mondada, 2016). One specific example is how the dynamics of conversational turn-taking are influenced not only by speech and linguistic cues, but also by visual signals such as manual gestures (Holler, Kendrick, & Levinson, 2018; Trujillo, Levinson, & Holler, 2021), body posture (Manrique & Enfield, 2015), and facial expression (Kendrick, 2015).

Recent work from both the behavioral and the neuroscientific domain has repeatedly demonstrated that visible speech and gestures can benefit speech comprehension in both clear and adverse listening conditions (e.g., Drijvers & Özyürek, 2017; Drijvers, Özyürek, & Jensen, 2018). Moreover, such visual signals can be used by an addressee, for example, to signal a lack of understanding and thus initiate a clarification attempt from the other speaker. Likewise, previous corpus studies and experimental work demonstrated that speakers modulate not only their speech, but also the kinematics of the visual signals they send when they are communicating in natural, noisy environments (Trujillo, Özyürek et al., 2021), and that utterances accompanied (vs. not accompanied) with manual gestures receive faster responses (Holler et al., 2018; Trujillo, Levinson, & Holler, 2021; ter Bekke, Drijvers, & Holle, 2020). More specifically, both the presence of gestures more generally, as well as greater kinematic salience of these gestures lead to both shorter gaps and overlaps between speakers (Trujillo, Levinson, & Holler, 2021), thus suggesting that visible gestures contribute to the tight timing seen in face-to-face conversations.

The timing of turn-taking is particularly interesting, as gaps between speakers are quite small, on the order of 200 ms (Stivers et al., 2009). This is remarkable because language production models predict these gaps to be closer to 500–600 ms, even for very short utterances (Levinson, 2016; Levinson & Torreira, 2015). To achieve this tight temporal coordination, speakers must prepare their utterance in parallel to listening (Heldner & Edlund, 2010; Levinson & Torreira, 2015). One way to achieve this is to predict upcoming speech, rather than waiting for the entire utterance to unfold. Visual information, such as manual gestures, could support this prediction process by providing early cues to what a speaker will refer to (ter Bekke, Drijvers, & Holle, 2020). Similarly, recent studies suggest that there is an inherent rhythmicity to conversational turn-taking that is regulated by multimodal signals in speech and bodily movement (Pouw & Holler, 2020; Pouw et al., 2021). These findings therefore suggest that turn-taking is not a fixed, rule-based system, but rather a dynamical system that emerges from the interplay of linguistic and (embodied) visual signals.

Multimodal signals can also facilitate alignment and common ground between speakers (Fusaroli & Tylén, 2016, see for a review: Rasenberg, Özyürek, & Dingemanse, 2020). For example, repeating lexical items between speakers can play a pivotal role in collaborative referencing, for example through priming (Pickering & Garrod, 2004) or incremental grounding of labels (e.g., Brennan & Clark, 1996; note that the meaning of grounding in this context is different from that in Table 1). Here, gestural information can convey semantic information that is complementary to the speech signal, aiding in collaborative referencing (Holler & Wilkin, 2011; Rasenberg, Özyürek, & Dingemanse, 2020). However, lexical alignment does not necessarily co-occur with gestural alignment (Oben & Brône, 2016), and it remains unclear whether and how alignment through visual bodily signals impacts conceptual alignment, or alignment between speakers at a neural level (e.g., brain-to-brain entrainment, but see Pan et al., 2020).

In sum, a theoretical and empirical framework that combines insights from multiple fields (e.g. linguistics, psychology, neuroscience) and methods (e.g., conversational analysis, corpus studies, experimental studies, kinematics) is needed to truly understand what the visual bodily signals add to producing and comprehending language in natural face-to-face conversations. Only such an integrative approach will unravel how visual bodily articulators contribute to language processing.

### Natural Language Data

At a global level of language, vast collections of natural language data in the form of large-scale corpora can serve as approximations of our language experience. Analyzing these corpora, especially distributional patterns of word usage, is another fruitful method for examining embodiment and grounding, showing that experience with language itself provides symbolic grounding for our conceptual system. Indeed, our everyday language experience inherently encodes an abundance of information about the world we live in, how we experience it, and how we interact with it (Johns & Jones, 2012; Louwerse, 2011).

In natural language, words are not distributed randomly. According to the distributional hypothesis (Harris, 1954), words with similar meanings occur in similar linguistic contexts (e.g., sentences, paragraphs, documents; Sahlgren, 2008). For instance, both *whale* and *dolphin* will often occur in the proximity of the words *ocean, sea, animal*, and maybe *fish* or *mammal*, but most likely not around *balcony, autumn*, or *e-mail*. Thus, the distributional patterns of *whale* and *dolphin* in natural language are very similar. According to a cognitive interpretation of the distributional hypothesis, distributional patterns and word meanings are inherently linked (Lenci, 2008). Specifically, meaning influences distributional information and, conversely, speakers use distributional information to learn the meaning of words. This is the core assumption underlying distributional semantic models (Günther, Rinaldi, & Marelli, 2019; Landauer & Dumais, 1997). **Distributional semantic models** (see Table 1) keep track of how often a given word occurs within given contexts in a corpus. The resulting distributions over contexts can be compared, leading to semantic similarity measures for word pairs. These semantic similarity measures have been demonstrated to predict, among many other phenomena, participant ratings of word similarity (Pereira et al., 2016) and semantic priming effects (Günther, Dudschig, & Kaup, 2016; Mandera, Keuleers, & Brysbaert, 2017).

As distributional semantic models are typically built from linguistic data, it is generally assumed that they would capture only abstract-symbolic knowledge (i.e., inter-connections in a fully self-contained system of abstract, amodal, and arbitrary linguistic symbols, see Glenberg, 2015; Glenberg & Robertson, 2000) and can therefore be contrasted with embodied and grounded knowledge derived from sensorimotor processes (i.e., concepts arising from direct experience with their referents; e.g., Borghesani & Piazza, 2017; Glenberg & Robertson, 2000). However, this perspective underestimates the knowledge that is implicitly encoded in natural language, which is not only abstract-symbolic. For instance, although individuals with congenital blindness lack first-hand sensory experience with colors, they are able to linguistically categorize colors and correctly assign colors to objects (Connolly, Gleitman, & Thompson-Schill, 2007; Shepard & Cooper, 1992). This surprising ability may be traced back to the distributional structure of language (Lewis, Zettersten, & Lupyan, 2019; Kim, Elli, & Bedny, 2019), which can serve as an indirect source of perception-relevant knowledge. Accordingly, a growing body of evidence indicates that experience with language can affect basic perceptual processing, including performance on visual recognition and discrimination tasks (Lupyan et al., 2020). In short, symbol grounding can occur by language that propagates embodied and grounded information through the conceptual system, and serves as experience by proxy (see **Symbol grounding-by-language** in Table 1).

A hypothetical individual whose experience is largely limited to language could still learn a surprising amount of information about the world. This has been demonstrated by several simulation studies, showing that distributional semantic models can accurately reproduce geographical information such as city distances (Louwerse & Zwaan, 2009). Additionally, the distributional structure of language has been found to replicate the organizational structure of the mental number line (i.e., distances between number words) and of the mental time line (i.e., distances between days of the week; Rinaldi & Marelli, 2020a; 2020b) and to predict human performance in symbolic number processing (Rinaldi, Parente, & Marelli, 2022). This argument even applies to the recently reported *subtraction neglect*, which refers to a preference to think of additive solutions instead of subtractive simplifications in a wide range of everyday tasks (Adams et al., 2021)—again, this bias is reflected in language statistics (Fischer et al., 2021). Thus, although it might appear counterintuitive, word distributions encode information beyond abstract-symbolic knowledge.

Natural language is not just a collection of random statements possible with the vocabulary and grammar of a given language. Instead, language is produced and used for specific communicative purposes. Typically, we talk about things that are relevant to us—our inner and outer experiences as we perceive and act in specific situations of our social and physical world. The resulting utterances will influence the distributional structure of the natural language data in language corpora. Distributional semantic models are able to pick up and represent this information. For example, in a model by Baroni, Dinu, and Kruszewski (2014), the most similar nouns to *tasty* were *pastas, bruschetta, tiramisu, tagine, antipasto*, and *taramasalata*. These things are not necessarily objectively the most similar concepts to tasty. Instead, when speakers describe their subjective experience, these words are often used in the same or similar contexts as *tasty*. Critically, language data serve as second-hand experience to other speakers, providing an opportunity to draw inferences. For example, even if you never had *taramasalata*, you can infer from the above that it is probably quite tasty. As elegantly described by Johnson-Laird (1983: 430), “a major function of language is thus to enable us to experience the world by proxy”. In this function as experience-by-proxy, language itself can serve as a means to establish symbol grounding (see **Symbol grounding problem** in Table 1), a scaffolding mechanism that allows us to simulate experiences we never had (for an early version of this argument, see Harnad, 1990).

Recent studies have provided empirical evidence for this symbol grounding-by-language process. For example, a study by Günther, Nguyen et al., (2020, described in the section *Sentences*) found that when participants learn novel concepts, these concepts are immediately grounded by language. Specifically, even when participants had learned new concepts purely linguistically, compatibility effects occurred, so that congruent movements were facilitated even though participants had never interacted with referents of these concepts (Günther, Nguyen et al., 2020). Similarly, Snefjella, Lana, and Kuperman (2020) found that initially meaningless novel words acquired emotional connotations (e.g., positive or negative valence) from the linguistic contexts they were presented in (e.g., describing the novel word referent as a plant living in *sunny green fields* vs. *muddy bogs*). Ecological evidence for symbol grounding-by-language comes also from natural language data (Günther, Petilli et al., 2020). In this study, a regression model was trained to map distributional vectors of image labels onto the corresponding image representations (based on computer vision models for image classification; Petilli et al., 2021; Vedaldi & Lenc, 2015). The resulting model was used to predict which images best represented words for which no image was available. When participants had to choose the image that best represented a given word, they preferred the model-predicted image over a random image, even for very abstract words (Günther, Petilli et al., 2020). This finding demonstrates that natural language systematically encodes the information required to estimate visual appearance of a concept, even in the absence of visual experience with that concept.

The reviewed findings highlight the use of computational characterizations based on proxies of linguistic and sensorimotor experiences. Models built on language distribution can provide quantitative predictions to be tested in empirical studies. These studies will supply a more fine-grained understanding of what is encoded in natural language. In sum, these findings indicate that language and sensorimotor experiences are fundamentally entwined. It may thus be time to leave behind strong dichotomous perspectives pitching the one against the other. Instead, it might be more fruitful to characterize language as an integrated, dynamic system, whose structure is shaped via experiences, both within a given source (i.e., in linguistic distributions) and between different sources (i.e., in the connections between language and sensorimotor systems; Johns & Jones, 2012).

## Discussion

Research on grounded, embodied, and situated cognition has revealed important insights across various granularity levels of language. Here, we reviewed evidence that, within specific boundary conditions, conceptual processing of words, sentences, and texts, involves the simulation of, for example, color, spatial location, sensory modality, and action direction. Moreover, we reviewed evidence for a finer level of linguistic granularity, showing that embodied and grounded processes can explain systematic associations between sublexical units of language and meaning, for example, why some vowels fit positive word meaning and others fit negative word meaning. Additionally, at a higher level of linguistic granularity, multimodal input can facilitate communication, and at the highest level, situated, embodied, and grounded knowledge is present in the information surrounding us, not only as explicit knowledge but even implicitly in which words typically occur in similar contexts.

On the sub-word and corpus levels, we are still at the stage of demonstrating that there are influences of embodied and grounded experiences; however, other levels have amassed a great number of findings, leading to a nuanced picture of generalizations and boundary conditions. For example, timing, either between different stimuli or between stimulus and response, seems to constitute an important boundary condition for embodied cognition on several levels of linguistic granularity (e.g., Borreggine & Kaschak, 2006; Boulenger et al., 2006; Connell & Lynott, 2012; Estes & Barsalou, 2018), whereas, somewhat surprisingly, previous experience with referents is not necessary for sensorimotor simulation (Günther et al., 2020). Moreover, research on the discourse processing level has progressed far enough to construct practical interventions, employing embodied cognition findings to improve reading comprehension (for interventions on different granularity levels, see Macedonia, 2014; Madan & Singhal, 2012; Reggin et al., 2022).

### Language as Independent and as Dependent Variable

Traditionally, and as illustrated in the present article, behavioral studies on embodied and grounded influences in language processing have mostly focused on various types of response times and error rates as their dependent measures. However, language does not just constitute a specific type of stimulus input; language is also directly observable human behavior. Thus, analyzing language itself *as a dependent variable* falls well within the scope of behavioral studies and should be more frequently employed to gain a more complete picture of how situated, embodied, and grounded processes influence language processing (for lab studies investigating language output produced by participants, see Rummer et al., 2014; Vogt, Kaup, & Abdel Rahman, 2021; Wu & Barsalou, 2009).

In the present article, this approach is exemplified in several sections: The studies on iconicity in both sign languages (e.g., Castillo, Fojo, & Aguirre, 2021) and spoken languages (e.g., Körner & Rummer, 2022) show how our language behavior in the form of the specific symbols we produce is grounded in sensorimotor experience. In addition, although the studies on natural language data presented here primarily focussed on how experiences can be *de*-coded from language (Günther, Petilli, et al., 2020; Lewis, Zettersten, & Lupyan, 2019; Rinaldi & Marelli, 2020a, 2020b), this is only possible because these experiences have been *en*-coded into language by other speakers in the first place. These examples provide a glimpse at the—still largely unrealized—potential of analyzing linguistic data from a grounded and embodied perspective as an avenue for psycholinguistic research (see also Gibbs, 2017; Johansson Falck & Gibbs, 2012; Winter, 2019). Taken together, these findings demonstrate that a dichotomy of language on the one hand and sensorimotor experiences on the other hand is at best blurry and at worst meaningless, as language is shaped by the grounded and embodied experiences of its speakers and transmits this information to language recipients.

### Comparing Language Processing to Number Processing

The present overview concentrated on grounded, embodied, and situated knowledge in language processing. For non-linguistic stimuli, similar effects have been observed, suggesting similar cognitive principles. Number processing is an especially interesting case in point. Similar to words, numbers (both as digits and as number words) have been found to orient spatial attention (Dehaene, Bossini, & Giraux, 1993). Moreover, spatial numerical associations (the SNARC effect) and spatial linguistic associations (the SLARC effect) might rest on the same processes, as biases in both tasks were found to be highly correlated (Abbondanza et al., 2021).

Just as numbers can be compared to words, arithmetic expressions can be compared to sentences in terms of their cognitive representations. Similar to sentences that have subjects, objects and verbs, arithmetic expressions like 2 × 2 + 3 also possess a syntactic structure that assigns roles to its elements. Supporting the similarity between language and arithmetic, bidirectional syntactic priming between language and arithmetic was observed (Scheepers & Sturt, 2014). Specifically, reading right-branching (e.g., 2 + 2 × 3) compared to left-branching (e.g., 2 × 2 + 3) arithmetic expressions facilitated the processing of right-branching (e.g., *bankrupt coffee dealer*) compared to left-branching (e.g., *organic coffee dealer*) linguistic compounds, and vice versa (Scheepers & Sturt, 2014). This finding supports the notion of a shared representation of numbers and language—not only at the word/number level of granularity but also at the sentence/arithmetic expression level of granularity. Similarly, the spatial association for numbers extends to addition and subtraction, with addition being associated to the right and subtraction to the left side of space (reviewed in Shaki, Pinhas, & Fischer, 2018).

However, in spite of these cross-domain similarities in terms of processing principles across knowledge levels, there are also differences between language and number processing. Whereas language processing involves linear scanning of elements, for arithmetic expressions, although starting with a left-biased attention (for participants with left-to-right reading direction), later parallel processing has been observed (Schneider et al., 2013). Similarly, the time course of spatial attention seems to be more task-dependent in arithmetic than in language processing. With written number presentation, a spatial bias occurred once an operator (e.g., plus or minus) was known (Liu et al., 2017). However, spontaneous eye movements during auditory presentation suggest that a second operand also needs to be known for spatial biases to occur (Masson, Letesson, & Pesenti, 2018; see also D’Ascenzo et al., 2020). Thus, it is not perfectly clear yet at what point in time spatial attention allocation in arithmetic expressions occurs.

### Evaluating the Strength of the Evidence

Experimental paradigms differ in the strength of evidence they provide for embodied cognition. Embodied cognition theories postulate sensorimotor simulations to be causally involved in conceptual processing—some theories even claim sensorimotor simulation completely constitutes conceptual processing, so that, according to strong embodied cognition theories, conceptual processing is nothing but simulation (e.g., Glenberg, 2015). However, critics argue that many findings cannot distinguish causal from epiphenomenal involvement, early from late involvement, and automatic from strategic involvement of sensorimotor simulation in conceptual processing. First, for brain imaging studies, finding that modality-specific brain regions were activated when reading words or sentences (e.g., Hauk, Johnsrude, & Pulvermüller, 2004), it has been argued that processing of modality-specific language might occur first, and simulation might ensue only after understanding. If this were the case, sensorimotor simulation would be only an epiphenomenon instead of a cause of understanding language (Mahon & Caramazza, 2008).

A second line of criticism is leveled against the interpretation of congruency effects as evidence for embodied cognition. Congruency paradigms are frequently used to examine sensorimotor simulation on a word or sentence level and, in contrast to brain-imaging studies, provide evidence for the causal involvement of sensorimotor processes. For example, in research on the action-sentence compatibility effect, quicker reactions when the literal movement direction matches (vs. mismatches) the movement direction implied by a sentence, has been interpreted as evidence that sentence comprehension rests on sensorimotor simulations (Glenberg & Kaschak, 2002). Alternatively, however, sensorimotor simulations might only affect later stages of processing, such as action preparation processes. Influences at both early stages (e.g., comprehension) and later stages (e.g., response preparation) would lead to compatibility effects, whereas only the former would be evidence for embodied cognition. Thus, critics argue, compatibility effects do not constitute strong evidence for the central tenet of embodied cognition that sensorimotor simulation is involved in understanding language (Ostarek & Huettig, 2019).

Third, several paradigms, prominent among them congruency paradigms, have been criticized for creating environments in which simulation facilitates task completion. Grounded or embodied influences have been found to be stronger, for example, when deciding on the spatial relation rather than the semantic relatedness of stimuli (Louwerse & Jeuniaux, 2010). Similarly, color congruency between implied object color and response button color has been found to be influenced by the proportion of items that were related to the response button color, so that for sentences a congruency effect only occurred when a large proportion of objects were associated with the response colors (Tsaregorodtseva et al., 2022). These findings are consistent with the idea that sensorimotor simulation might not be automatic but instead be only strategically used when its use facilitates task completion (Machery, 2007). This kind of task-dependent or strategic employment of simulation would be different from the automatic sensorimotor simulation postulated by many embodied cognition theories (e.g., Barsalou, 1999).

These criticisms have been addressed in different ways by different empirical paradigms. Causal instead of epiphenomenal involvement has been demonstrated using congruency paradigms, where faster reactions when simulation and response are congruent compared to incongruent (see sections on *Word* and *Sentence* processing). Similarly, studies that show performance improvements when simulation is possible (vs. impaired, either by a long-lasting disorder or concurrent interference; e.g., Boulenger et al., 2008; Niedenthal et al., 2009) or multimodal cues (e.g., gestures in addition to voice) are given (vs. not given) during conversation (Drijvers & Özyürek, 2017) provide additional evidence for the causal role postulated by embodied cognition theories. Additionally, these impairment and multimodality paradigms speak for the involvement of sensorimotor processes during conceptual processing as opposed to involvement only during other phases of task completion (see also Ostarek & Bottini, 2021). Moreover, brain imaging studies with a higher temporal resolution observed somatotopic brain activity as early as 80 ms after stimulus presentation (Shtyrov et al., 2014; see also Carota, Moseley, & Pulvermüller, 2012; van Elk et al., 2010) indicating that brain activity in modality-specific brain areas occurs very early. Very early activity rather argues against sensorimotor simulation being strategically used or being involved only during later processing states.

Concerning the question of whether simulation is automatic or strategically employed, the sheer number of boundary conditions for simulation effects for various sensory, emotional, and motor properties strongly suggests that sensorimotor simulation is task-dependent (for similar reasoning, see Ostarek & Huettig, 2019). However, finding boundary conditions does not mean that the process underlying a phenomenon is only strategically active; instead the process could be active by default and in most normal circumstances. To determine whether grounded and embodied processes are active in many situations in real life, examining naturally-produced language is especially useful, because it is not produced in contexts designed to elicit grounded, embodied, or situated cognition and has a high degree of ecological validity. In the present research, we examined two kinds of natural language data for evidence of grounded and embodied cognition. First, on the sub-word level, we described how embodied and grounded processes could have shaped the very words we use, as evidenced by iconicity phenomena. Second, on the natural language data level, we described studies showing that grounded and embodied knowledge is encoded in distributional patterns in general-purpose natural language corpora (i.e., corpora that were not constructed to investigate questions about grounded, embodied, or situated cognition). Taken together, these studies demonstrate how grounding and embodiment influence language use across many different situations in real life. Thus, even if grounded and embodied processes are not always active, they are frequent in normal situations—so frequent as to shape our language itself.

## Conclusion

Perhaps the strongest conclusion that we can draw is that no one task, paradigm, or approach is going to answer all questions about embodied cognition. This consensus article has reviewed a variety of behavioral approaches useful for investigating embodiment and grounding in language. But we have also referred to other approaches, such as brain imaging and neuropsychology, that are needed to carefully test predictions and fully develop explanations. We believe that we are far from exhausting the important contributions that multiple methods can make to fleshing out and testing embodiment theories.

## Footnotes

For bears in general, the typical color is brown; but for polar bears, the typical color is white. For our example in the main text, whether brown or white is the simulated color in readers’ minds depends on their integration of the available context information.

## References

[1] Abbondanza, M., Rinaldi, L., Foppolo, F., & Marelli, M. (2021). The mental representation of nonnumerical quantifiers: The spatial-linguistic association of response codes (SLARC) effect. Journal of Experimental Psychology: Learning, Memory, and Cognition, 47(12), 2021–2028. DOI: 10.1037/xlm0001037 34582237

[2] Adams, A. M., Glenberg, A. M., & Restrepo, M. A. (2019). Embodied reading in a transparent orthography. Learning and Instruction, 62, 27–36. DOI: 10.1016/j.learninstruc.2019.03.003

[3] Adams, G. S., Converse, B. A., Hales, A. H., & Klotz, L. E. (2021). People systematically overlook subtractive changes. Nature, 592, 258–261. DOI: 10.1038/s41586-021-03380-y 33828317

[4] Alisedo, G., Pastor, I., Ayala, D., Bacigalupo, E., Lassalle, L., & Costa, F. (2007). Diccionario bilingüe de lengua de señas uruguaya/español. Asociación de Sordos de Uruguay.

[5] Banks, B., Borghi, A. M., Fargier, R., Fini, C., Jonauskaite, D., Mazzuca, C., Montalti, M., Villani, C., & Woodin, G. (2022). Consensus Paper: Current Perspectives on Abstract Concepts and Future Research Directions. [Manuscript submitted for publication]. 10.5334/joc.238 PMC10573588 37841672

[6] Baroni, M., Dinu, G., & Kruszewski, G. (2014). Don’t count, predict! A systematic comparison of context-counting vs. context-predicting semantic vectors. In Proceedings of the 52nd Annual Meeting of the Association for Computational Linguistics (pp. 238–247). DOI: 10.3115/v1/P14-1023

[7] Barsalou, L. W. (1999). Perceptual symbol systems. Behavioral and Brain Sciences, 22(4), 577–660. DOI: 10.1017/S0140525X99002149 11301525

[8] Barsalou, L. W. (2008). Grounded cognition. Annual Review of Psychology, 59, 617–645. DOI: 10.1146/annurev.psych.59.103006.093639 17705682

[9] Baus, C., Carreiras, M., & Emmorey, K. (2013). When does iconicity in sign language matter? Language and Cognitive Processes, 28, 261–271. DOI: 10.1080/01690965.2011.620374 23543899 PMC3608132

[10] Bechtold, L., Cosper, S. H., Malyshevskaya, A., Montefinese, M., Morucci, P., Niccolai, V., Repetto, C., Zappa, A., & Shtyrov, Y. (2022). Brain Signatures of Embodied Semantics and Language: A Consensus Paper. [Manuscript submitted for publication]. 10.5334/joc.237 PMC10573703 37841669

[11] Berenhaus, M., Oakhill, J., & Rusted, J. (2015). When kids act out: A comparison of embodied methods to improve children’s memory for a story. Journal of Research in Reading, 38(4), 331–343. DOI: 10.1111/1467-9817.12039

[12] Bergen, B. K. (2004). The psychological reality of phonaesthemes. Language, 80(3), 290–311. DOI: 10.1353/lan.2004.0056

[13] Bergen, B. K. (2015). Embodiment, simulation and meaning. In N. Riemer (Ed.), The Routledge Handbook of Semantics (pp. 158–173). Routledge. DOI: 10.4324/9781315685533

[14] Bhalla, M., & Proffitt, D. R. (1999). Visual–motor recalibration in geographical slant perception. Journal of Experimental Psychology: Human Perception and Performance, 25(4), 1076–1096. DOI: 10.1037/0096-1523.25.4.1076 10464946

[15] Blasi, D. E., Wichmann, S., Hammarström, H., Stadler, P. F., & Christiansen, M. H. (2016). Sound–meaning association biases evidenced across thousands of languages. Proceedings of the National Academy of Sciences, 113(39), 10818–10823. DOI: 10.1073/pnas.1605782113 PMC5047153 27621455

[16] Borghesani, V., & Piazza, M. (2017). The neuro-cognitive representations of symbols: The case of concrete words. Neuropsychologia, 105, 4–17. DOI: 10.1016/j.neuropsychologia.2017.06.026 28648571

[17] Borghi, A. M., Barca, L., Binkofski, F., Castelfranchi, C., Pezzulo, G., & Tummolini, L. (2019). Words as social tools: Language, sociality and inner grounding in abstract concepts. Physics of Life Reviews, 29, 120–153. DOI: 10.1016/j.plrev.2018.12.001 30573377

[18] Boroditsky, L. (2000). Metaphoric structuring: Understanding time through spatial metaphors. Cognition, 75(1), 1–28. DOI: 10.1016/S0010-0277(99)00073-6 10815775

[19] Borreggine, K. L., & Kaschak, M. P. (2006). The action–sentence compatibility effect: It’s all in the timing. Cognitive Science, 30(6), 1097–1112. DOI: 10.1207/s15516709cog0000_91 21702848

[20] Boulenger, V., Mechtouff, L., Thobois, S., Broussolle, E., Jeannerod, M., & Nazir, T. A. (2008). Word processing in Parkinson’s disease is impaired for action verbs but not for concrete nouns. Neuropsychologia, 46(2), 743–756. DOI: 10.1016/j.neuropsychologia.2007.10.007 18037143

[21] Boulenger, V., Roy, A. C., Paulignan, Y., Deprez, V., Jeannerod, M., & Nazir, T. A. (2006). Cross-talk between language processes and overt motor behavior in the first 200 ms of processing. Journal of Cognitive Neurosciene, 18(10), 1607–1615. DOI: 10.1162/jocn.2006.18.10.1607 17014366

[22] Brennan, S. E., & Clark, H. H. (1996). Conceptual pacts and lexical choice in conversation. Journal of Experimental Psychology: Learning, Memory, and Cognition, 22(6), 1482–1493. DOI: 10.1037/0278-7393.22.6.1482 8921603

[23] Carota, F., Moseley, R., & Pulvermüller, F. (2012). Body-part-specific representations of semantic noun categories. Journal of Cognitive Neuroscience, 24(6), 1492–1509. DOI: 10.1162/jocn_a_00219 22390464

[24] Casasanto, D. (2009). Embodiment of abstract concepts: Good and bad in right- and left-handers. Journal of Experimental Psychology: General, 138(3), 351–367. DOI: 10.1037/a0015854 19653795

[25] Caselli, N. K., Sehyr, Z. S., Cohen-Goldberg, A. M., & Emmorey, K. (2017). ASL-LEX: A lexical database of American Sign Language. Behavior Research Methods, 49(2), 784–801. DOI: 10.3758/s13428-016-0742-0 27193158 PMC5116008

[26] Castillo, M., Fojo, A., & Aguirre, R. (2021). Distribution of unidimensional space in the LSU time lexicon. In Proceedings of the 43rd Annual Meeting of the Cognitive Science Society (p. 3259). https://escholarship.org/uc/item/5qn2w1p2

[27] Chasteen, A. L., Burdzy, D. C., & Pratt, J. (2010). Thinking of God moves attention. Neuropsychologia, 48(2), 627–630. DOI: 10.1016/j.neuropsychologia.2009.09.029 19804790

[28] Connell, L. (2007). Representing object colour in language comprehension. Cognition, 102(3), 476–485. DOI: 10.1016/j.cognition.2006.02.009 16616075

[29] Connell, L., & Lynott, D. (2009). Is a bear white in the woods? Parallel representation of implied object color during language comprehension. Psychonomic Bulletin & Review, 16(3), 573–577. DOI: 10.3758/PBR.16.3.573 19451387

[30] Connell, L., & Lynott, D. (2012). When does perception facilitate or interfere with conceptual processing? The effect of attentional modulation. Frontiers in Psychology, 3, Article 474. DOI: 10.3389/fpsyg.2012.00474 PMC3487424 23130012

[31] Connolly, A. C., Gleitman, L. R., & Thompson-Schill, S. L. (2007). Effect of congenital blindness on the semantic representation of some everyday concepts. Proceedings of the National Academy of Sciences, 104(20), 8241–8246. DOI: 10.1073/pnas.0702812104 PMC1895936 17483447

[32] Ćwiek, A., Fuchs, S., Draxler, C., Asu, E. L., Dediu, D., Hiovain, K., Kawahara, S., Koutalidis, S., Krifka, M., Lippus, P., Lupyan, G., Oh, G. E., Paul, J., Petrone, C., Ridouane, R., Reiter, S., Schümchen, N., Szalontai, A., Ünal-Logacev, Ö., Zeller, J., Perlman, M., & Winter, B. (2021). The bouba/kiki effect is robust across cultures and writing systems. Philosophical Transactions of the Royal Society B, 377(1841), Article 20200390. DOI: 10.1098/rstb.2020.0390 PMC8591387 34775818

[33] D’Ascenzo, S., Lugli, L., Nicoletti, R., & Fischer, M. H. (2020). Assessing orienting of attention to understand the time course of mental calculation. Cognitive Processing, 21(4), 493–500. DOI: 10.1007/s10339-020-00970-y 32696298

[34] de Koning, B. B., Wassenburg, S. I., Bos, L. T., & van der Schoot, M. (2017). Mental simulation of four visual object properties: Similarities and differences as assessed by the sentence–picture verification task. Journal of Cognitive Psychology, 29(4), 420–432. DOI: 10.1080/20445911.2017.1281283

[35] de Vega, M., Moreno, V., & Castillo, D. (2013). The comprehension of action-related sentences may cause interference rather than facilitation on matching actions. Psychological Research, 77(1), 20–30. DOI: 10.1007/s00426-011-0356-1 21755368

[36] Dehaene, S., Bossini, S., & Giraux, P. (1993). The mental representation of parity and number magnitude. Journal of Experimental Psychology: General, 122(3), 371–396. DOI: 10.1037/0096-3445.122.3.371

[37] Dingemanse, M. (2012). Advances in the cross-linguistic study of ideophones. Language and Linguistics Compass, 6(10), 654–672. DOI: 10.1002/lnc3.361

[38] Dingemanse, M., Blasi, D. E., Lupyan, G., Christiansen, M. H., & Monaghan, P. (2015). Arbitrariness, iconicity, and systematicity in language. Trends in Cognitive Sciences, 19(10), 603–615. DOI: 10.1016/j.tics.2015.07.013 26412098

[39] Dooley, E. (2021). SNARC effect for verbal probability quantifiers in economic decision making task. [Master’s thesis, Berlin School of Mind & Brain, Humboldt University]. The Open Science Framework. https://osf.io/uq5zr/?view_only=11cbe1a11a8c401892ee391c00a6a92e

[40] Drijvers, L., & Özyürek, A. (2017). Visual context enhanced: The joint contribution of iconic gestures and visible speech to degraded speech comprehension. Journal of Speech, Language, and Hearing Research, 60(1), 212–222. DOI: 10.1044/2016_JSLHR-H-16-0101 27960196

[41] Drijvers, L., Özyürek, A., & Jensen, O. (2018). Hearing and seeing meaning in noise: Alpha, beta, and gamma oscillations predict gestural enhancement of degraded speech comprehension. Human Brain Mapping, 39(5), 2075–2087. DOI: 10.1002/hbm.23987 29380945 PMC5947738

[42] Emmorey, K. (2001). Space on hand: The exploitation of signing space to illustrate abstract thought. In M. Gattis (Ed.), Spatial schemas and abstract thought (pp. 147–174). MIT Press.

[43] Estes, Z., & Barsalou, L. W. (2018). A comprehensive meta-analysis of spatial interference from linguistic cues: Beyond Petrova et al. (2018). Psychological Science, 29(9), 1558–1564. DOI: 10.1177/0956797618794131 30113254

[44] Estes, Z., Verges, M., & Barsalou, L. W. (2008). Head up, foot down: Object words orient attention to the objects’ typical location. Psychological Science, 19(2), 93–97. DOI: 10.1111/j.1467-9280.2008.02051.x 18271853

[45] Fischer, M. H. (2012). A hierarchical view of grounded, embodied and situated numerical cognition. Cognitive Processing, 13(S1), S161–S164. DOI: 10.1007/s10339-012-0477-5 22802036

[46] Fischer, M. H., & Brugger, P. (2011). When digits help digits: Spatial–numerical associations point to finger counting as prime example of embodied cognition. Frontiers in Psychology, 2, Article 260. DOI: 10.3389/fpsyg.2011.00260 PMC3198540 22028696

[47] Fischer, M. H., & Shaki, S. (2018). Number concepts: Abstract and embodied. Philosophical Transactions of the Royal Society B: Biological Sciences, 373(1752), Article 20170125. DOI: 10.1098/rstb.2017.0125 PMC6015824 29914993

[48] Fischer, M. H., Winter, B., Felisatti, A., Myachykov, A., Mende, M. A., & Shaki, S. (2021). More Instructions Make Fewer Subtractions. Frontiers in Psychology, 12, Article 20616. DOI: 10.3389/fpsyg.2021.720616 PMC8506214 34650481

[49] Fischer, M. H., & Zwaan, R. A. (2008). Embodied language: A review of the role of the motor system in language comprehension. Quarterly Journal of Experimental Psychology, 61(6), 825–850. DOI: 10.1080/17470210701623605 18470815

[50] Frak, V., Nazir, T., Goyette, M., Cohen, H., & Jeannerod, M. (2010). Grip force is part of the semantic representation of manual action verbs. PloS one, 5(3), Article e9728. DOI: 10.1371/journal.pone.0009728 20300535 PMC2838801

[51] Fusaroli, R., & Tylén, K. (2016). Investigating conversational dynamics: Interactive alignment, interpersonal synergy, and collective task performance. Cognitive Science, 40(1), 145–171. DOI: 10.1111/cogs.12251 25988263

[52] Gallese, V., & Lakoff, G. (2005). The brain’s concepts: The role of the sensory-motor system in conceptual knowledge. Cognitive Neuropsychology, 22(3–4), 455–479. DOI: 10.1080/02643290442000310 21038261

[53] Gambrell, L. B., & Jawitz, P. B. (1993). Mental imagery, text illustrations, and children’s story comprehension and recall. Reading Research Quarterly, 265–276. DOI: 10.2307/747998

[54] García, A. M., & Ibáñez, A. (2016). A touch with words: Dynamic synergies between manual actions and language. Neuroscience & Biobehavioral Reviews, 68, 59–95. DOI: 10.1016/j.neubiorev.2016.04.022 27189784

[55] Garrido, M. V., & Godinho, S. (2021). When vowels make us smile: The influence of articulatory feedback in judgments of warmth and competence. Cognition and Emotion, 35(5), 837–843. DOI: 10.1080/02699931.2021.1900076 33745414

[56] Gianelli, C., Farnè, A., Salemme, R., Jeannerod, M., & Roy, A. C. (2011). The agent is right: When motor embodied cognition is space-dependent. PLoS one, 6(9). Article e25036. DOI: 10.1371/journal.pone.0025036 21966407 PMC3179480

[57] Gibbs, R. W. Jr., (2017). Embodiment. In B. Dancygier (Ed.), The Cambridge Handbook of Cognitive Linguistics (pp. 449–462). Cambridge University Press. DOI: 10.1017/9781316339732

[58] Glenberg, A. M. (2015). Few believe the world is flat: How embodiment is changing the scientific understanding of cognition. Canadian Journal of Experimental Psychology, 69, 165–171. DOI: 10.1037/cep0000056 26010024

[59] Glenberg, A. M., & Gallese, V. (2012). Action-based language: A theory of language acquisition, comprehension, and production. Cortex, 48(7), 905–922. DOI: 10.1016/j.cortex.2011.04.010 21601842

[60] Glenberg, A. M., Gutierrez, T., Levin, J. R., Japuntich, S., & Kaschak, M. P. (2004). Activity and imagined activity can enhance young children’s reading comprehension. Journal of Educational Psychology, 96(3), 424–436. DOI: 10.1037/0022-0663.96.3.424

[61] Glenberg, A. M., & Kaschak, M. P. (2002). Grounding language in action. Psychonomic Bulletin & Review, 9(3), 558–565. DOI: 10.3758/BF03196313 12412897

[62] Glenberg, A. M., & Robertson, D. A. (1999). Indexical understanding of instructions. Discourse Processes, 28(1), 1–26. DOI: 10.1080/01638539909545067

[63] Glenberg, A. M., & Robertson, D. A. (2000). Symbol grounding and meaning: A comparison of high-dimensional and embodied theories of meaning. Journal of Memory and Language, 43, 379–401. DOI: 10.1006/jmla.2000.2714

[64] Goldberg, R. F., Perfetti, C. A., & Schneider, W. (2006). Perceptual knowledge retrieval activates sensory brain regions. Journal of Neuroscience, 26(18), 4917–4921. DOI: 10.1523/JNEUROSCI.5389-05.2006 16672666 PMC6674166

[65] Gómez, L., & Glenberg, A. M. (2022). Embodied classroom activities for vocabulary acquisition. In S. L. Macrine, & J. Fugate (Eds.), Movement Matters: How Embodied Cognition Informs Teaching and Learning (pp. 77–90). MIT Press.

[66] Gozli, D. G., Chasteen, A. L., & Pratt, J. (2013). The cost and benefit of implicit spatial cues for visual attention. Journal of Experimental Psychology: General, 142(4), 1028–1046. DOI: 10.1037/a0030362 23106305

[67] Günther, F., Dudschig, C., & Kaup, B. (2016). Latent semantic analysis cosines as a cognitive similarity measure: Evidence from priming studies. The Quarterly Journal of Experimental Psychology, 69, 626–653. DOI: 10.1080/17470218.2015.1038280 25952009

[68] Günther, F., Nguyen, T., Chen, L., Dudschig, C., Kaup, B., & Glenberg, A. M. (2020). Immediate sensorimotor grounding of novel concepts learned from language alone. Journal of Memory and Language, 115, Article 104172. DOI: 10.1016/j.jml.2020.104172

[69] Günther, F., Petilli, M. A., Vergallito, A., & Marelli, M. (2020). Images of the unseen: Extrapolating visual representations for abstract and concrete words in a data-driven computational model. Psychological Research. Advance online publication. DOI: 10.1007/s00426-020-01429-7 PMC9674750 33180152

[70] Günther, F., Rinaldi, L., & Marelli, M. (2019). Vector-space models of semantic representation from a cognitive perspective: A discussion of common misconceptions. Perspectives on Psychological Science, 14, 1006–1033. DOI: 10.1177/1745691619861372 31505121

[71] Hald, L. A., Marshall, J.-A., Janssen, D. P., & Garnham, A. (2011). Switching modalities in a sentence verification task: ERP evidence for embodied language processing. Frontiers in Psychology, 2, Article 45. DOI: 10.3389/fpsyg.2011.00045 PMC3132671 21779254

[72] Harnad, S. (1990). The symbol grounding problem. Physica D: Nonlinear Phenomena, 42(1–3), 335–346. DOI: 10.1016/0167-2789(90)90087-6

[73] Harris, Z. (1954). Distributional structure. Word, 10(2–3), 146–162. DOI: 10.1080/00437956.1954.11659520

[74] Hauk, O., Johnsrude, I., & Pulvermüller, F. (2004). Somatotopic representation of action words in human motor and premotor cortex. Neuron, 41(2), 301–307. DOI: 10.1016/S0896-6273(03)00838-9 14741110

[75] Havas, D. A., Glenberg, A. M., & Rinck, M. (2007). Emotion simulation during language comprehension. Psychonomic Bulletin & Review, 14(3), 436–441. DOI: 10.3758/BF03194085 17874584

[76] Havas, D. H., Glenberg, A. M., Gutowski, K., Lucarelli, M., & Davidson, R. (2010). Cosmetic use of Botulinum Toxin-A affects processing of emotional language. Psychological Science, 21, 895–900. DOI: 10.1177/0956797610374742 20548056 PMC3070188

[77] Heldner, M., & Edlund, J. (2010). Pauses, gaps and overlaps in conversations. Journal of Phonetics, 38(4), 555–568. DOI: 10.1016/j.wocn.2010.08.002

[78] Hockett, C. F. (1963). The problem of universals in language. In J. H. Greenberg (Ed.), Universals of language (pp. 1–29). MIT Press.

[79] Holler, J., Kendrick, K. H., & Levinson, S. C. (2018). Processing language in face-to-face conversation: Questions with gestures get faster responses. Psychonomic Bulletin & Review, 25(5), 1900–1908. DOI: 10.3758/s13423-017-1363-z 28887798

[80] Holler, J., & Wilkin, K. (2011). Co-speech gesture mimicry in the process of collaborative referring during face-to-face dialogue. Journal of Nonverbal Behavior, 35(2), 133–153. DOI: 10.1007/s10919-011-0105-6

[81] Hommel, B., Pratt, J., Colzato, L., & Godijn, R. (2001). Symbolic control of visual attention. Psychological Science, 12(5), 360–365. DOI: 10.1111/1467-9280.00367 11554667

[82] Horchak, O. V., & Garrido, M. V. (2022). Simulating background settings during spoken and written sentence comprehension. Psychonomic Bulletin & Review. DOI: 10.3758/s13423-022-02061-9 PMC8821844 35132579

[83] Horchak, O. V., Giger, J. C., Cabral, M., & Pochwatko, G. (2014). From demonstration to theory in embodied language comprehension: A review. Cognitive Systems Research, 29, 66–85. DOI: 10.1016/j.cogsys.2013.09.002

[84] Horchak, O. V., Giger, J. C., & Pochwatko, G. (2014). Discourse comprehension and simulation of positive emotions. Psicologica: International Journal of Methodology and Experimental Psychology, 35(1), 17–37.

[85] Huettig, F., & Altmann, G. T. (2011). Looking at anything that is green when hearing “frog”: How object surface colour and stored object colour knowledge influence language-mediated overt attention. Quarterly Journal of Experimental Psychology, 64(1), 122–145. DOI: 10.1080/17470218.2010.481474 20521211

[86] Huettig, F., Guerra, E., & Helo, A. (2020). Towards understanding the task dependency of embodied language processing: The influence of colour during language-vision interactions. Journal of Cognition, 3(1), Article 41. DOI: 10.5334/joc.135 PMC7583718 33134815

[87] Ibáñez, A., Kühne, K., Miklashevsky, A., Monaco, E., Muraki, E., Ranzini, M., Speed, L. J., & Tuena, C. (2022). The importance of considering individual differences and context to understand embodied language processes [Manuscript submitted for publication].

[88] Imai, M., & Kita, S. (2014). The sound symbolism bootstrapping hypothesis for language acquisition and language evolution. Philosophical Transactions of the Royal Society B: Biological Sciences, 369(1651), Article 20130298. DOI: 10.1098/rstb.2013.0298 PMC4123677 25092666

[89] Jeannerod, M. (2001). Neural simulation of action: A unifying mechanism for motor cognition. Neuroimage, 14(1), S103–S109. DOI: 10.1006/nimg.2001.0832 11373140

[90] Johansson Falck, M., & Gibbs, R. W. Jr., (2012). Embodied motivations for metaphorical meanings. Cognitive Linguistics, 23(2), 251–272. DOI: 10.1515/cog-2012-0008

[91] Johns, B. T., & Jones, M. N. (2012). Perceptual inference through global lexical similarity. Topics in Cognitive Science, 4(1), 103–120. DOI: 10.1111/j.1756-8765.2011.01176.x 22253184

[92] Johnson-Laird, P. N. (1983). Mental models: Towards a cognitive science of language, inference, and consciousness. Harvard University Press.

[93] Kaschak, M. P., & Borreggine, K. L. (2008). Temporal dynamics of the action-sentence compatibility effect. Quarterly Journal of Experimental Psychology, 61(6), 883–895. DOI: 10.1080/17470210701623852 PMC4612619 18470819

[94] Kendrick, K. H. (2015). Other-initiated repair in English. Open Linguistics, 1(1), 164–190. DOI: 10.2478/opli-2014-0009

[95] Kim, J. S., Elli, G. V., & Bedny, M. (2019). Knowledge of animal appearance among sighted and blind adults. Proceedings of the National Academy of Sciences, 116(23), 11213–11222. DOI: 10.1073/pnas.1900952116 PMC6561279 31113884

[96] Kita, S. (1997). Two-dimensional semantic analysis of Japanese mimetics. Linguistics, 35(2), 379–415. DOI: 10.1515/ling.1997.35.2.379

[97] Klatzky, R. L., Pellegrino, J. W., McCloskey, B. P., & Doherty, S. (1989). Can you squeeze a tomato? The role of motor representations in semantic sensibility judgments. Journal of Memory and Language, 28(1), 56–77. DOI: 10.1016/0749-596X(89)90028-4

[98] Köhler, W. (1929). Gestalt psychology. Liveright.

[99] Körner, A., & Rummer, R. (2022). Articulation contributes to valence sound symbolism. Journal of Experimental Psychology: General, 151(5), 1107–1114. DOI: 10.1037/xge0001124 34694857

[100] Körner, A., Topolinski, S., & Strack, F. (2015). Routes to embodiment. Frontiers in Psychology, 6, Article 940. DOI: 10.3389/fpsyg.2015.00940 PMC4488596 26191033

[101] Kuhnke, P., Kiefer, M., & Hartwigsen, G. (2020). Task-dependent recruitment of modality-specific and multimodal regions during conceptual processing. Cerebral Cortex, 30(7), 3938–3959. DOI: 10.1093/cercor/bhaa010 32219378 PMC7264643

[102] Lakoff, G., & Johnson, M. (1980). Metaphors we live by. University of Chicago Press.

[103] Landauer, T. K., & Dumais, S. T. (1997). A solution to Plato’s problem: The latent semantic analysis theory of acquisition, induction, and representation of knowledge. Psychological Review, 104, 211–240. DOI: 10.1037/0033-295X.104.2.211

[104] Lenci, A. (2008). Distributional semantics in linguistic and cognitive research. Italian Journal of Linguistics, 20(1), 1–31.

[105] Levinson, S. C. (2016). Turn-taking in human communication: Origins and implications for language processing. Trends in Cognitive Sciences, 20(1), 6–14. DOI: 10.1016/j.tics.2015.10.010 26651245

[106] Levinson, S. C., & Torreira, F. (2015). Timing in turn-taking and its implications for processing models of language. Frontiers in Psychology, 6, Article 731. DOI: 10.3389/fpsyg.2015.00731 PMC4464110 26124727

[107] Lewis, M., Zettersten, M., & Lupyan, G. (2019). Distributional semantics as a source of visual knowledge. Proceedings of the National Academy of Sciences, 116(39), 19237–19238. DOI: 10.1073/pnas.1910148116 PMC6765286 31488726

[108] Liu, D., Cai, D., Verguts, T., & Chen, Q. (2017). The time course of spatial attention shifts in elementary arithmetic. Scientific Reports, 7, Article 921. DOI: 10.1038/s41598-017-01037-3 PMC5430428 28424467

[109] Louwerse, M. M., & Connell, L. (2011). A taste of words: Linguistic context and perceptual simulation predict the modality of words. Cognitive Science, 35(2), 381–398. DOI: 10.1111/j.1551-6709.2010.01157.x 21429005

[110] Louwerse, M. M. (2011). Symbol interdependency in symbolic and embodied cognition. Topics in Cognitive Science, 3(2), 273–302. DOI: 10.1111/j.1756-8765.2010.01106.x 25164297

[111] Louwerse, M. M., & Jeuniaux, P. (2010). The linguistic and embodied nature of conceptual processing. Cognition, 114(1), 96–104. DOI: 10.1016/j.cognition.2009.09.002 19818435

[112] Louwerse, M. M., & Zwaan, R. A. (2009). Language encodes geographical information. Cognitive Science, 33(1), 51–73. DOI: 10.1111/j.1551-6709.2008.01003.x 21585463

[113] Lukas, S., Philipp, A. M., & Koch, I. (2010). Switching attention between modalities: Further evidence for visual dominance. Psychological Research, 74(3), 255–267. DOI: 10.1007/s00426-009-0246-y 19517132

[114] Lupyan, G., Abdel Rahman, R., Boroditsky, L., & Clark, A. (2020). Effects of language on visual perception. Trends in Cognitive Sciences, 24(11), 930–944. DOI: 10.1016/j.tics.2020.08.005 33012687

[115] Lynott, D., & Connell, L. (2009). Modality exclusivity norms for 423 object properties. Behavior Research Methods, 41(2), 558–564. DOI: 10.3758/BRM.41.2.558 19363198

[116] Macedonia, M. (2014). Bringing back the body into the mind: Gestures enhance word learning in foreign language. Frontiers in Psychology, 5, Article 1467. DOI: 10.3389/fpsyg.2014.01467 PMC4260465 25538671

[117] Machery, E. (2007). Concept empiricism: A methodological critique. Cognition, 104(1), 19–46. DOI: 10.1016/j.cognition.2006.05.002 16814274

[118] Madan, C. R., & Singhal, A. (2012). Using actions to enhance memory: Effects of enactment, gestures, and exercise on human memory. Frontiers in Psychology, 3, Article 507. DOI: 10.3389/fpsyg.2012.00507 PMC3536268 23293612

[119] Mahon, B. Z., & Caramazza, A. (2008). A critical look at the embodied cognition hypothesis and a new proposal for grounding conceptual content. Journal of Physiology-Paris, 102(1–3), 59–70. DOI: 10.1016/j.jphysparis.2008.03.004 18448316

[120] Mandera, P., Keuleers, E., & Brysbaert, M. (2017). Explaining human performance in psycholinguistic tasks with models of semantic similarity based on prediction and counting: A review and empirical validation. Journal of Memory and Language, 92, 57–78. DOI: 10.1016/j.jml.2016.04.001

[121] Mannaert, L. N. H., Dijkstra, K., & Zwaan, R. A. (2017). Is color an integral part of a rich mental simulation? Memory & Cognition, 45(6), 974–982. DOI: 10.3758/s13421-017-0708-1 28439728 PMC5529485

[122] Manrique, E., & Enfield, N. (2015). Suspending the next turn as a form of repair initiation: Evidence from Argentine sign language. Frontiers in Psychology, 6, Article 1326. DOI: 10.3389/fpsyg.2015.01326 PMC4569752 26441710

[123] Masson, M., Letesson, C., & Pesenti, M. (2018). Time course of overt attentional shifts in mental arithmetic: Evidence from gaze metrics. Quarterly Journal of Experimental Psychology, 71(4), 1009–1019. DOI: 10.1080/17470218.2017.1318931 28399712

[124] McCrink, K., Dehaene, S., & Dehaene-Lambertz, G. (2007). Moving along the number line: Operational momentum in non-symbolic arithmetic. Perception & Psychophysics, 69(8), 1324–1333. DOI: 10.3758/BF03192949 18078224

[125] Meteyard, L., Cuadrado, S. R., Bahrami, B., & Vigliocco, G. (2012). Coming of age: A review of embodiment and the neuroscience of semantics. Cortex, 48(7), 788–804. DOI: 10.1016/j.cortex.2010.11.002 21163473

[126] Mioni, G., Fischer, M. H., & Shaki, S. (2021). Heuristics and biases in the mental manipulation of magnitudes: Evidence from length and time production. Quarterly Journal of Experimental Psychology, 74(3), 536–547. DOI: 10.1177/1747021820967663 33063598

[127] Monaghan, P., Shillcock, R. C., Christiansen, M. H., & Kirby, S. (2014). How arbitrary is language? Philosophical Transactions of the Royal Society B: Biological Sciences, 369(1651), Article 20130299. DOI: 10.1098/rstb.2013.0299 PMC4123678 25092667

[128] Mondada, L. (2016). Challenges of multimodality: Language and the body in social interaction. Journal of Sociolinguistics, 20(3), 336–366. DOI: 10.1111/josl.1_12177

[129] Morey, R. D., Kaschak, M. P., Díez-Álamo, A. M., Glenberg, A. M., Zwaan, R. A., Lakens, D., … & Ziv-Crispel, N. (2022). A pre-registered, multi-lab non-replication of the action-sentence compatibility effect (ACE). Psychonomic Bulletin & Review, 29(2), 613–626. DOI: 10.3758/s13423-021-01927-8 34755319 PMC9038876

[130] Murgiano, M., Motamedi, Y., & Vigliocco, G. (2021). Situating language in the real-world: The role of multimodal iconicity and indexicality. Journal of Cognition, 4(1), Article 38. DOI: 10.5334/joc.113 PMC8396123 34514309

[131] Myachykov, A., Scheepers, C., Fischer, M. H., & Kessler, K. (2014). TEST: A tropic, embodied, and situated theory of cognition. Topics in Cognitive Science, 6(3), 442–460. DOI: 10.1111/tops.12024 23616259

[132] Naor-Raz, G., Tarr, M. J., & Kersten, D. (2003). Is color an intrinsic property of object representation? Perception, 32(6), 667–680. DOI: 10.1068/p5050 12892428

[133] Niedenthal, P. M., Winkielman, P., Mondillon, L., & Vermeulen, N. (2009). Embodiment of emotion concepts. Journal of Personality and Social Psychology, 96(6), 1120–1136. DOI: 10.1037/a0015574 19469591

[134] Núñez, R., & Cooperrider, K. (2013). The tangle of space and time in human cognition. Trends in Cognitive Sciences, 17(5), 220–229. DOI: 10.1016/j.tics.2013.03.008 23608363

[135] Oakhill, J. V., Cain, K., & Bryant, P. E. (2003). The dissociation of word reading and text comprehension: Evidence from component skills. Language and Cognitive Processes, 18(4), 443–468. DOI: 10.1080/01690960344000008

[136] Oben, B., & Brône, G. (2016). Explaining interactive alignment: A multimodal and multifactorial account. Journal of Pragmatics, 104, 32–51. DOI: 10.1016/j.pragma.2016.07.002

[137] Ohala, J. (1994). The frequency code underlies the sound-symbolic use of voice pitch. In L. Hinton, J. Nichols, & J. Ohala (Eds.), Sound Symbolism (pp. 325–347). Cambridge University Press. DOI: 10.1017/CBO9780511751806.022

[138] Ostarek, M., & Bottini, R. (2021). Towards strong inference in research on embodiment: Possibilities and limitations of causal paradigms. Journal of Cognition, 4(1), Article 5. DOI: 10.5334/joc.139 PMC7792456 33506171

[139] Ostarek, M., & Huettig, F. (2019). Six challenges for embodiment research. Current Directions in Psychological Science, 28(6), 593–599. DOI: 10.1177/0963721419866441

[140] Ostarek, M., Joosen, D., Ishag, A., de Nijs, M., & Huettig, F. (2019). Are visual processes causally involved in “perceptual simulation” effects in the sentence-picture verification task? Cognition, 182, 84–94. DOI: 10.1016/j.cognition.2018.08.017 30219635

[141] Pan, Y., Dikker, S., Goldstein, P., Zhu, Y., Yang, C., & Hu, Y. (2020). Instructor-learner brain coupling discriminates between instructional approaches and predicts learning. NeuroImage, 211, Article 116657. DOI: 10.1016/j.neuroimage.2020.116657 32068165

[142] Papesh, M. H. (2015). Just out of reach: On the reliability of the action-sentence compatibility effect. Journal of Experimental Psychology: General, 144(6), e116–e141. DOI: 10.1037/xge0000125 26595844 PMC4662055

[143] Parise, C. V., & Spence, C. (2012). Audiovisual crossmodal correspondences and sound symbolism: A study using the Implicit Association Test. Experimental Brain Research, 220(3–4), 319–333. DOI: 10.1007/s00221-012-3140-6 22706551

[144] Pecher, D., Zeelenberg, R., & Barsalou, L. W. (2003). Verifying different-modality properties for concepts produces switching costs. Psychological Science, 14(2), 119–124. DOI: 10.1111/1467-9280.t01-1-01429 12661672

[145] Pecher, D., Zeelenberg, R., & Barsalou, L. W. (2004). Sensorimotor simulations underlie conceptual representations: Modality-specific effects of prior activation. Psychonomic Bulletin & Review, 11(1), 164–167. DOI: 10.3758/BF03206477 15117003

[146] Pereira, F., Gershman, S., Ritter, S., & Botvinick, M. (2016). A comparative evaluation of off-the-shelf distributed semantic representations for modelling behavioural data. Cognitive Neuropsychology, 33(3–4), 175–190. DOI: 10.1080/02643294.2016.1176907 27686110

[147] Perniss, P., Thompson, R., & Vigliocco, G. (2010). Iconicity as a general property of language: Evidence from spoken and signed languages. Frontiers in Psychology, 1, Article 227. DOI: 10.3389/fpsyg.2010.00227 PMC3153832 21833282

[148] Petilli, M. A., Günther, F., Vergallito, A., Ciapparelli, M., & Marelli, M. (2021). Data-driven computational models reveal perceptual simulation in word comprehension. Journal of Memory and Language, 117, Article 104194. DOI: 10.1016/j.jml.2020.104194

[149] Petrova, A., Navarrete, E., Suitner, C., Sulpizio, S., Reynolds, M., Job, R., & Peressotti, F. (2018). Spatial congruency effects exist, just not for words: Looking into Estes, Verges, and Barsalou (2008). Psychological Science, 29(7), 1195–1199. DOI: 10.1177/0956797617728127 29874144

[150] Pezzulo, G., Barsalou, L. W., Cangelosi, A., Fischer, M. H., Spivey, M., & McRae, K. (2013). Computational grounded cognition: A new alliance between grounded cognition and computational modeling. Frontiers in Psychology, 3, Article 612. DOI: 10.3389/fpsyg.2012.00612 PMC3551279 23346065

[151] Pfau, R., Steinbach, M., & Woll, B. (2012). Sign language. De Gruyter Mouton. DOI: 10.1515/9783110261325

[152] Pickering, M. J., & Garrod, S. (2004). Toward a mechanistic psychology of dialogue. Behavioral and Brain Sciences, 27(2), 169–226. DOI: 10.1017/S0140525X04000056 15595235

[153] Platonova, O., & Miklashevsky, A. (2022). Warm + fuzzy: Perceptual semantics can be activated even during surface lexical processing. Manuscript in preparation. DOI: 10.17605/OSF.IO/A85H7 39946601

[154] Posner, M. I. (1980). Orienting of attention. Quarterly Journal of Experimental Psychology, 32(1), 3–25. DOI: 10.1080/00335558008248231 7367577

[155] Pouw, W., & Holler, J. (2020). Timing in conversation is dynamically adjusted turn by turn: Evidence for lag-1 negatively autocorrelated turn taking times in telephone conversation. PsyArXiv preprint. DOI: 10.31234/osf.io/b98da

[156] Pouw, W., Proksch, S., Drijvers, L., Gamba, M., Holler, J., Kello, C., Schaefer, R. S., & Wiggins, G. A. (2021). Multilevel rhythms in multimodal communication. Philosophical Transactions of the Royal Society B: Biological Sciences, 376(1835), Article 20200334. DOI: 10.1098/rstb.2020.0334 PMC8380971 34420378

[157] Pulvermüller, F., Shtyrov, Y., & Hauk, O. (2009). Understanding in an instant: Neurophysiological evidence for mechanistic language circuits in the brain. Brain and Language, 110(2), 81–94. DOI: 10.1016/j.bandl.2008.12.001 19664815 PMC2734884

[158] Rasenberg, M., Özyürek, A., & Dingemanse, M. (2020). Alignment in multimodal interaction: An integrative framework. Cognitive Science, 44(11), Article e12911. DOI: 10.1111/cogs.12911 33124090 PMC7685147

[159] Reggin, L. D., Gómez Franco, L. E., Horchak, O. V., Labrecque, D., Lana, N., Lorenzini, I., Rio, L., & Vigliocco, G. (2022). Language is not acquired in the lab but in the real world: The role of embodied and situated cognition. [Manuscript submitted for publication].

[160] Rinaldi, L., & Marelli, M. (2020a). The use of number words in natural language obeys Weber’s law. Journal of Experimental Psychology: General, 149(7), 1215–1230. DOI: 10.1037/xge0000715 31789571

[161] Rinaldi, L., & Marelli, M. (2020b). Maps and space are entangled with language experience. Trends in Cognitive Sciences, 24(11), 853–855. DOI: 10.1016/j.tics.2020.07.009 32972827

[162] Rinaldi, L., Parente, L., & Marelli, M. (2022). Toward a unified account of nonsymbolic and symbolic representations of number: Insights from a combined psychophysical-computational approach. Psychonomic Bulletin & Review, 29(3), 985–994. DOI: 10.3758/s13423-021-02043-3 34918278

[163] Robinson, M. D., & Thomas, L. E. (2021). Introduction to Embodied Psychology: Thinking, Feeling, and Acting. In M. D. Robinson & L. E. Thomas (Eds.), Handbook of Embodied Psychology (pp. 1–19). Springer. DOI: 10.1007/978-3-030-78471-3_1

[164] Rueschemeyer, S. A., Glenberg, A. M., Kaschak, M., Mueller, K., & Friederici, A. (2010). Top-down and bottom-up contributions to understanding sentences describing objects in motion. Frontiers in Psychology, 1, Article 183. DOI: 10.3389/fpsyg.2010.00183 PMC3153793 21833244

[165] Rummer, R., & Schweppe, J. (2019). Talking emotions: Vowel selection in fictional names depends on the emotional valence of the to-be-named faces and objects. Cognition and Emotion, 33(3), 404–416. DOI: 10.1080/02699931.2018.1456406 29658373

[166] Rummer, R., Schweppe, J., Schlegelmilch, R., & Grice, M. (2014). Mood is linked to vowel type: The role of articulatory movements. Emotion, 14(2), 246–250. DOI: 10.1037/a0035752 24708505

[167] Sahlgren, M. (2008). The distributional hypothesis. Italian Journal of Linguistics, 20, 33–53.

[168] Sapir, E. (1929). A study in phonetic symbolism. Journal of Experimental Psychology, 12(3), 225–239. DOI: 10.1037/h0070931

[169] Scerrati, E., Baroni, G., Borghi, A. M., Galatolo, R., Lugli, L., & Nicoletti, R. (2015). The modality-switch effect: Visually and aurally presented prime sentences activate our senses. Frontiers in Psychology, 6, Article 1668. DOI: 10.3389/fpsyg.2015.01668 PMC4627474 26579049

[170] Scerrati, E., Lugli, L., Nicoletti, R., & Borghi, A. M. (2017). The multilevel modality-switch effect: What happens when we see the bees buzzing and hear the diamonds glistening. Psychonomic Bulletin & Review, 24(3), 798–803. DOI: 10.3758/s13423-016-1150-2 27542801

[171] Schank, R. C., & Abelson R. P. (1977). Scripts, plans, goals and understanding: An inquiry into human knowledge structures. Lawrence Erlbaum.

[172] Scheepers, C., & Sturt, P. (2014). Bidirectional syntactic priming across cognitive domains: From arithmetic to language and back. Quarterly Journal of Experimental Psychology, 67(8), 1643–1654. DOI: 10.1080/17470218.2013.873815 24328811

[173] Schembri, A., Fenlon, J., Rentelis, R., Reynolds, S., & Cormier, K. (2013). Building the British sign language corpus. Language Documentation & Conservation, 7, 136–154. http://hdl.handle.net/10125/4592

[174] Schneider, E., Maruyama, M., Dehaene, S., & Sigman, M. (2013). Eye gaze reveals a fast, parallel extraction of the syntax of arithmetic formulas. Cognition, 125(3), 475–490. DOI: 10.1016/j.cognition.2012.06.015 22921187

[175] Shaki, S., Pinhas, M., & Fischer, M. H. (2018). Heuristics and biases in mental arithmetic: Revisiting and reversing operational momentum. Thinking and Reasoning, 24(2), 138–156. DOI: 10.1080/13546783.2017.1348987

[176] Shapiro, L., & Spaulding, S. (2021). Embodied Cognition. In E. N. Zalta (Ed.), The Stanford Encyclopedia of Philosophy (Winter 2021 ed.). Stanford University. https://plato.stanford.edu/archives/win2021/entries/embodied-cognition/

[177] Shebani, Z., & Pulvermüller, F. (2018). Flexibility in language action interaction: The influence of movement type. Frontiers in Human Neuroscience, 12, Article 252. DOI: 10.3389/fnhum.2018.00252 PMC6026896 29988612

[178] Shepard, R. N., & Cooper, L. A. (1992). Representation of colors in the blind, color-blind, and normally sighted. Psychological Science, 3(2), 97–104. DOI: 10.1111/j.1467-9280.1992.tb00006.x

[179] Shtyrov, Y., Butorina, A., Nikolaeva, A., & Stroganova, T. (2014). Automatic ultrarapid activation and inhibition of cortical motor systems in spoken word comprehension. Proceedings of the National Academy of Sciences, 111(18), E1918–E1923. DOI: 10.1073/pnas.1323158111 PMC4020094 24753617

[180] Sidhu, D. M., & Pexman, P. M. (2018). Five mechanisms of sound symbolic association. Psychonomic Bulletin & Review, 25(5), 1619–1643. DOI: 10.3758/s13423-017-1361-1 28840520

[181] Sinte, A. (2013). Expression of time in French Belgian Sign Language (LSFB). In L. Meurant, A. Sinte, M. van Herreweghe & M. Vermeerbergen (Eds.), Sign language research, uses and practices: Crossing views on theoretical and applied sign language linguistics (pp. 205–235). De Gruyter Mouton; Ishara Press. DOI: 10.1515/9781614511472.205

[182] Snefjella, B., Lana, N., & Kuperman, V. (2020). How emotion is learned: Semantic learning of novel words in emotional contexts. Journal of Memory and Language, 115, Article 104171. DOI: 10.1016/j.jml.2020.104171

[183] Stivers, T., Enfield, N. J., Brown, P., Englert, C., Hayashi, M., Heinemann, T., Hoymann, G., Rossano, F., Ruiter, J. P. de, Yoon, K.-E., & Levinson, S. C. (2009). Universals and cultural variation in turn-taking in conversation. Proceedings of the National Academy of Sciences, 106(26), 10587–10592. DOI: 10.1073/pnas.0903616106 PMC2705608 19553212

[184] ter Bekke, M., Drijvers, L., & Holler, J. (2020). The predictive potential of hand gestures during conversation: An investigation of the timing of gestures in relation to speech. PsyArXiV preprint. DOI: 10.31234/osf.io/b5zq7 38279899

[185] Thompson, P. D., & Estes, Z. (2011). Sound symbolic naming of novel objects is a graded function. Quarterly Journal of Experimental Psychology, 64(12), 2392–2404. DOI: 10.1080/17470218.2011.605898 21895561

[186] Thompson, R. L., Vinson, D. P., & Vigliocco, G. (2009). The link between form and meaning in American Sign Language: Lexical processing effects. Journal of Experimental Psychology: Learning, Memory, and Cognition, 35(2), 550–557. DOI: 10.1037/a0014547 19271866 PMC3667647

[187] Thorndyke, P. W. (1977). Cognitive structures in comprehension and memory of narrative discourse. Cognitive Psychology, 9(1), 77–110. DOI: 10.1016/0010-0285(77)90005-6

[188] Trujillo, J. P., Levinson, S. C., & Holler, J. (2021). Visual information in computer-mediated interaction matters: Investigating the association between the availability of gesture and turn transition timing in conversation. In M. Kurosu (Ed.), Human-Computer Interaction. Design and User Experience Case Studies (pp. 643–657). Springer. DOI: 10.1007/978-3-030-78468-3_44

[189] Trujillo, J. P., Özyürek, A., Holler, J., & Drijvers, L. (2021). Speakers exhibit a multimodal Lombard effect in noise. Scientific Reports, 11(1), Article 16721. DOI: 10.1038/s41598-021-95791-0 PMC8373897 34408178

[190] Tsaregorodtseva, O., Frazier, L., Stolterfoht, B., & Kaup, B. (2022). Does language activate the sensorimotor properties of the entities it refers to and, if so, under what circumstances? Manuscript in preparation. DOI: 10.17605/OSF.IO/N7M9U

[191] Vainio, L., & Vainio, M. (2021). Sound-action symbolism. Frontiers in Psychology, 12, Article 718700. DOI: 10.3389/fpsyg.2021.718700 PMC8476841 34594278

[192] van Elk, M., van Schie, H. T., Zwaan, R. A., & Bekkering, H. (2010). The functional role of motor activation in language processing: Motor cortical oscillations support lexical-semantic retrieval. NeuroImage, 50(2), 665–677. DOI: 10.1016/j.neuroimage.2009.12.123 20060478

[193] Vedaldi, A., & Lenc, K. (2015). MatConvNet: Convolutional neural networks for Matlab. In Proceedings of the 23rd ACM International Conference on Multimedia (pp. 689–692). DOI: 10.1145/2733373.2807412

[194] Vigliocco, G., Vinson, D. P., Lewis, W., & Garrett, M. F. (2004). Representing the meanings of object and action words: The featural and unitary semantic space hypothesis. Cognitive Psychology, 48(4), 422–488. DOI: 10.1016/j.cogpsych.2003.09.001 15099798

[195] Vogt, A., Kaup, B., & Abdel Rahman, R. (2021). Experience-driven meaning affects lexical choices during language production. PsyArXiV preprint. DOI: 10.31234/osf.io/ru7ms PMC10280667 36062350

[196] Wasner, M., Moeller, K., Fischer, M. H., & Nuerk, H. C. (2014). Aspects of situated cognition in embodied numerosity: The case of finger counting habits. Cognitive Processing, 15, 317–328. DOI: 10.1007/s10339-014-0599-z 24435616

[197] Whalen, D. H., & Levitt, A. G. (1995). The universality of intrinsic F_0_ of vowels. Journal of Phonetics, 23(3), 349–366. DOI: 10.1016/S0095-4470(95)80165-0

[198] Winter, A., Dudschig, C., & Kaup, B. (2021). The action-sentence compatibility effect (ACE) a benchmark finding for embodiment: A meta-analysis. PsyArXiV preprint. DOI: 10.31234/osf.io/wfpz7 36103797

[199] Winter, B. (2019). Sensory linguistics: Language, perception and metaphor. John Benjamins. DOI: 10.1075/celcr.20

[200] Winter, B., Perlman, M., Perry, L. K., & Lupyan, G. (2017). Which words are most iconic? Iconicity in English sensory words. Interaction Studies, 18(3), 443–464. DOI: 10.1075/is.18.3.07win

[201] Wu, L. L., & Barsalou, L. W. (2009). Perceptual simulation in conceptual combination: Evidence from property generation. Acta Psychologica, 132(2), 173–189. DOI: 10.1016/j.actpsy.2009.02.002 19298949

[202] Yee, E., Ahmed, S. Z., & Thompson-Schill, S. L. (2012). Colorless green ideas (can) prime furiously. Psychological Science, 23(4), 364–369. DOI: 10.1177/0956797611430691 22374271 PMC4152373

[203] Yu, C. S., McBeath, M. K., & Glenberg, A. M. (2021). The gleam-glum effect: /i:/ versus /λ/ phonemes generically carry emotional valence. Journal of Experimental Psychology: Learning, Memory, and Cognition, 47(7), 1173–1185. DOI: 10.1037/xlm0001017 34694842

[204] Zwaan, R. A. (2021). Two challenges to “embodied cognition” research and how to overcome them. Journal of Cognition, 4(1), Article 14. DOI: 10.5334/joc.151 PMC7894377 33634231

[205] Zwaan, R. A., & Pecher, D. (2012). Revisiting mental simulation in language comprehension: Six replication attempts. PLoS one, 7(12), Article e51382. DOI: 10.1371/journal.pone.0051382 23300547 PMC3530580

[206] Zwaan, R. A., & Radvansky, G. A. (1998). Situation models in language comprehension and memory. Psychological Bulletin, 123(2), 162–185. DOI: 10.1037/0033-2909.123.2.162 9522683

